# A metamodel for mobile forensics investigation domain

**DOI:** 10.1371/journal.pone.0176223

**Published:** 2017-04-26

**Authors:** Abdulalem Ali, Shukor Abd Razak, Siti Hajar Othman, Arafat Mohammed, Faisal Saeed

**Affiliations:** 1 Faculty of Computing, Universiti Teknologi Malaysia, Skudai,Johor, Malaysia; 2 Faculty of Engineering and Information Technology, Taiz University, Taiz, Yemen; University of Texas at San Antonio, UNITED STATES

## Abstract

With the rapid development of technology, mobile phones have become an essential tool in terms of crime fighting and criminal investigation. However, many mobile forensics investigators face difficulties with the investigation process in their domain. These difficulties are due to the heavy reliance of the forensics field on knowledge which, although a valuable resource, is scattered and widely dispersed. The wide dispersion of mobile forensics knowledge not only makes investigation difficult for new investigators, resulting in substantial waste of time, but also leads to ambiguity in the concepts and terminologies of the mobile forensics domain. This paper developed an approach for mobile forensics domain based on metamodeling. The developed approach contributes to identify common concepts of mobile forensics through a development of the Mobile Forensics Metamodel (MFM). In addion, it contributes to simplifying the investigation process and enables investigation teams to capture and reuse specialized forensic knowledge, thereby supporting the training and knowledge management activities. Furthermore, it reduces the difficulty and ambiguity in the mobile forensics domain. A validation process was performed to ensure the completeness and correctness of the MFM. The validation was conducted using two techniques for improvements and adjustments to the metamodel. The last version of the adjusted metamodel was named MFM 1.2.

## 1. Introduction

**Fig 1:**
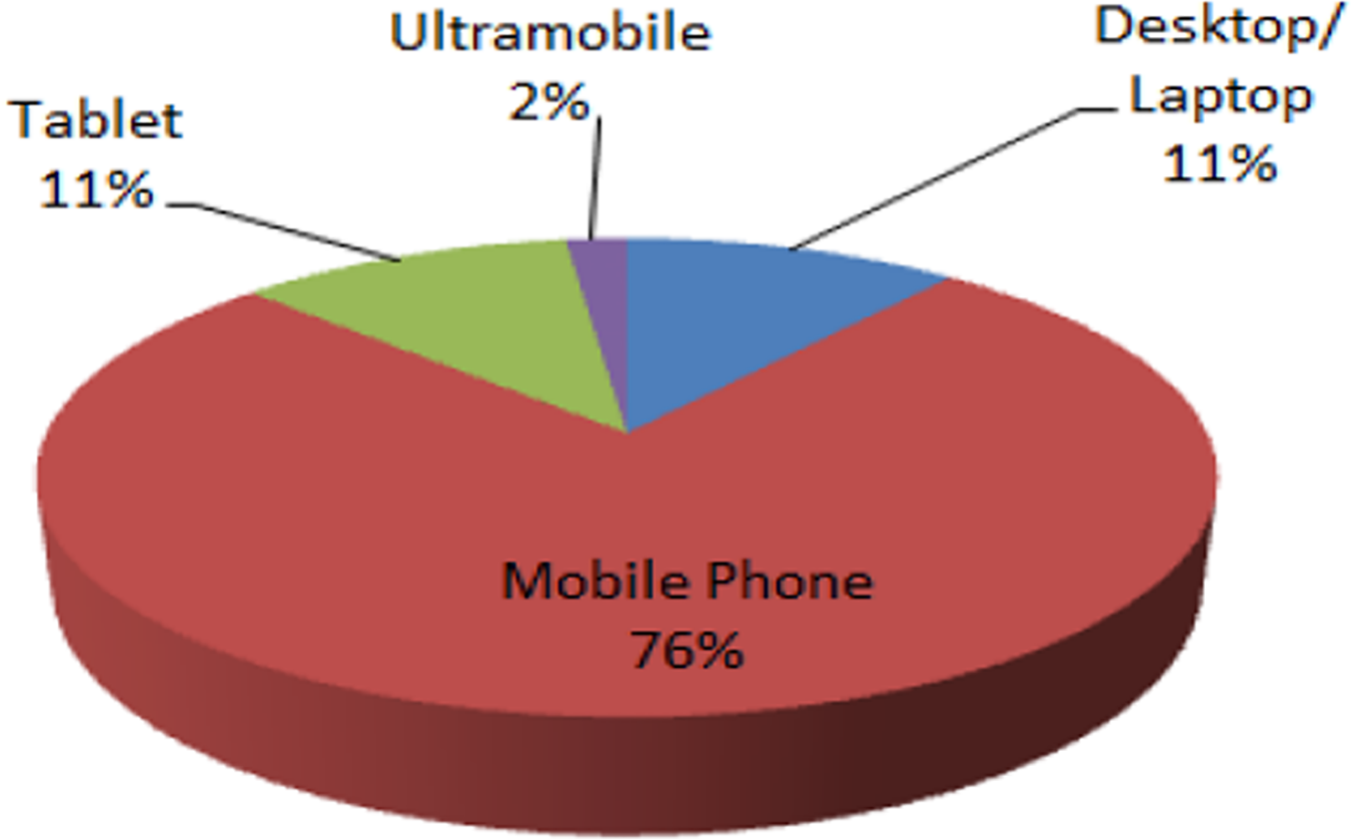
Worldwide device shipments in year 2014[3].

The worldwide use of mobile phone devices is increasing daily. Ericsson President and CEO Hans Vestberg expects that by 2020, 50 billion mobile phones will be connected to the web as compared to five billion now [1]. This confirms an earlier prediction that by 2020 mobile phones will be the primary devices of digital communication [2]. Fig 1 shows that 76 percent of the devices used in 2014 were mobile phones[3]. According to a recent report by Patrik Cerwall (2015), the number of mobile phone users in Q1 2015 was around 7.2 billion, which equals the World’s population[4].

Mobile device forensics is considered a new field compared to other digital forensics such as computer and database forensics. According to authors in [5], Mobile Forensics (MF) is a branch of digital forensics relating to the recovery of digital evidence from a mobile device under forensically sound conditions. The phrase 'mobile device' often applies to mobile phones. However, these devices are currently used for many other activities in our daily lives, for instance, checking e-mails, taking photos, browsing the Internet, business transactions, location data and much more. In contrast to these productive activities, mobile phone crime is on the rise, and cybercrime is now moving to mobile phone devices. For instance, committing fraud via email, harassment through text messages, distribution of child pornography, terrorism and selling drugs. MF has many interacting elements, including people, authority, investigation teams, resources, procedures and policy. The sophistication of the crimes and the variety of mobile phone devices used in these offenses are becoming major challenges to the investigators[6]. In addition, the volume of data and complexity of investigation are among the major issues in MF[7].

Furthermore, the investigators may not have a clear view of which potential evidence to start the investigation with. Previous studies have mostly discussed mobile forensics only in data acquisition terms and only in a problem solving scenario, as a subset to computer forensics. These studies did not take mobile forensics beyond the paradigm that is known as computer forensics. Additionally, they have not addressed the elements of MF comprehensively, and the previous research in the MF domain did not focus on modeling the case domain information involved in investigations [8]. The existing mobile forensic models are not based on any metamodels but rather constitute proprietary solutions, mainly focused on frameworks and other aspects of models. Metamodeling has been promoted by the efforts of the Object Management Group (OMG) [9]. This paper develops a Mobile Forensic Metamodel (MFM) in order to clarify all the necessary activities required by investigators for conducting their task. In addition, it creates a unified view of mobile forensic in the form of a metamodel that can be seen as a language for this domain. A metamodeling approach is applied to ensure that the metamodel which is the outcome is complete and consistent.

The rest of this paper is organized as follows: the background of MF summarized in Section 2; Section 3 presents and describes the development process of our mobile forensic metamodel, based on a metamodeling approach; finally, the conclusion is presented in Section 4.

## 2. Background

The rapid change in the technology of mobile phones has provided opportunities for criminal activities. The crimes conducted through mobile phones include fraud, drugs and pornography, as discussed in [10] which indicated that these crimes are growing with the increase in numbers of mobile devices. According to the National Institute of Justice [11], many digital crimes are committed annually through mobile phone devices due to the proliferation of these devices in most countries. Thus, mobile phone devices contain a great deal of digital evidence for digital investigation processes[12]. The purpose of extracting digital evidence from mobile phone devices is to use it in court proceedings, as these devices are now frequently used in criminal activities [13]. The extracted evidence from mobile phones has played a significant role in forensics investigation in recent years and many murderer convictions have been partly based on evidence gathered from the mobile phones of the perpetrators or their victims [14]. For instance, mobile phone evidence was used in the prosecution of Ian Huntley who killed two girls, and was also used to locate and arrest suspects in the failed London car bomb attacks in 2007 [12]. Some of the types of crimes conducted through the use of mobiles and the evidence sources contained in the mobile devices are shown in Table 1.

**Table 1:** Mobile phone digital crimes [11].

Crime	Description	Evidence Source
**Harassment**	By sending any type of (text, sexual, photo, video) messages that contain harassing and threatening words.	-Message box—Calendars. - Chat logs—Gallery photos/videos. - Address books—History logs file.
**Trafficking Drugs**	Criminals using mobile phones to distribute drugs and coordinate activities between them.	-Messages box—Calendars. -Gallery photos—Call history. -Contact lists—Cell site locations. -GPS-Electronic money transfers.
**Terrorism**	Dangerous actions against civilians to achieve political, organization goals by using mobile phones as a bomb (e.g.: Mumbai terrorist attack 2008, and commuter trains in Madrid in 2004, or using the mobile to coordinate activities and share information).	-Cell site locations—Call history. -Messages box—Calendars. -Gallery photos—GPS. -Electronic money transfers.
**Fraud**	Using mobile banking app features to send fake information that looks like an original to the victims.	- Internet history logs. —Call history.

The rapid proliferation of mobile phones on the market caused a demand for forensic examination of the devices, which could not be met by existing computer forensic techniques. Much research has been conducted in the MF domain. While some studies have discussed MF in general devices, the majority of previous studies were concerned with Smartphone forensics. A study in [15] tested and analyzed data remnants for instant messaging from Facebook and Skype to identify evidence from these data. However, validated frameworks and methods to extract mobile phone data are practically non-existent [16]. The rapid development in mobile phone devices has caused difficulties to designing a single forensic tool or standards specific to one platform [12]. Furthermore, the lack of hardware, software and standardization in mobile phone devices are one of the significant difficulties in the MF domain [17]. This fact makes investigation process a hard task. It is also easy to tamper with digital evidence in mobile phones through overwritten or remote commands received from the wireless network [18].

Moreover, the lack of standardization is a major issue in the field of MF. Advanced development in technology, as well as the variety of mobile devices and OSs are making the procedure of developing a common framework or standardization model difficult [17, 19, 20]. In addition, as stated in [21], that the major issue in mobile phone investigation is that there is no standard forensic model nor any standard process for the forensic examination of smart phones. Research by Hoog concluded that digital forensic investigators and security engineers have faced difficulties dealing with mobile phone crimes due to their lack of knowledge management[22].

Additionally, it has been suggested that members of the legal profession need to increase their level of understanding and knowledge of mobile phone forensic terminology, techniques and procedures [13]. Moreover, it has been claimed that a major issue in law enforcement agencies in many countries is the lack of knowledge management [23]. Therefore, forensic investigators are facing difficult challenges when conducting the forensic investigation processes related to digital crimes, particularly for mobile phones. In a recent NIST Mobile Forensics Workshop (2014) [24] conducted by researchers in the MF domain, all the issues related to MF domain were discussed. It was indicated that investigators are struggling with the MF domain because they do not have sufficient knowledge, training and education related to the proper seizure procedures for mobile devices, the appropriate transport procedures and proper forensic examination and analysis [24]. Furthermore, while a number of digital forensic process models have been developed by various organizations worldwide, there are no agreed forensic investigation and legislative delegation procedures to adhere to, especially in the case of dealing with mobile devices with the latest technologies [25]. Recently, several studies have been focused on mobile forensics. However, these were mostly concerned with cloud forensics [26–38].

## 3. Mobile forensic metamodel

**Fig 2:**
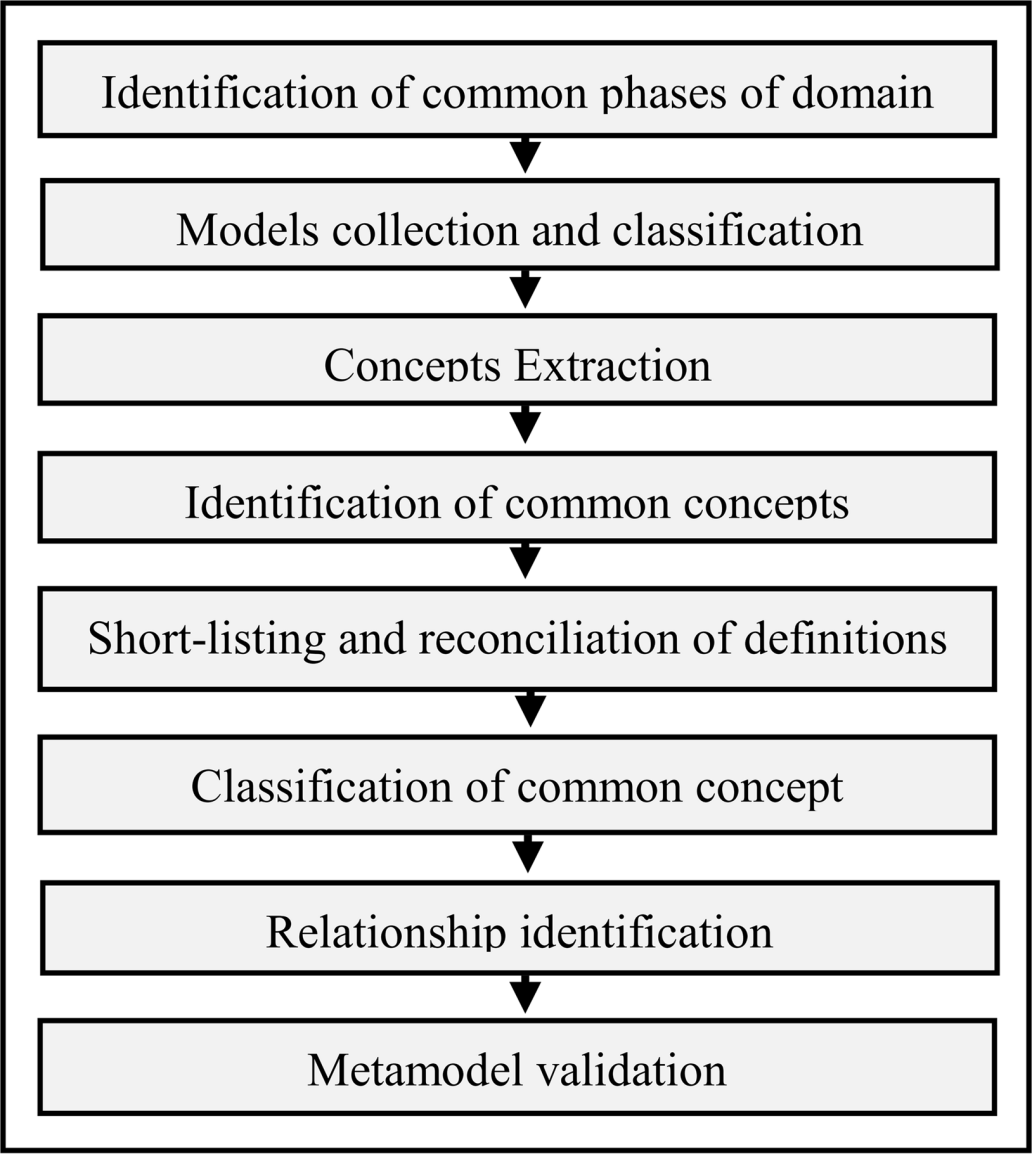
Metamodeling process.

In this paper, the authors use a metamodeling approach to identify the common concepts of the MF domain. This approach has been promoted by the efforts of the Object Management Group (OMG) to create interoperable, reusable and components. This is an activity to generalize a domain through collecting all the domain concepts and partitioning the domain problems into sub-domain-problems [39]. Through this approach we developed our metamodel for MF. Thus, a metamodel is a special kind of a model: It identifies domain features and related concepts (like any other model) but is created with the intent to formally describe the semantics underpinning a formal modelling language. Without a metamodel, the semantics of domain models can be ambiguous [40]. Many previous studies have used metamodeling approach for managing knowledge of domain. The study reported in [39] used this approach to develop a generic metamodel for Multi Agent System (MAS). They used 6-steps to develop their metamodel. Later, a metamodel for managing disaster management knowledge was developed [40], using an 8-step metamodeling creation process. Moreover, the study in [41] used 7-step of metamodeling process to design a comprehensive and general purpose metamodel for metacognition that support artificial intelligence systems. To develop MFM, we used an 8-step metamodeling process adapted from [39], [40] and [41], which are the works most closely related to this study, and which present a thorough and structured process for identifying major concepts and their relationships. Fig 2 illustrates these steps.

### 3.1 Identification of common phases of domain

The purpose of identifying the common phases of the domain is to facilitate the extraction concepts in the domain. According to [5, 42], the common phases of MF include Preservation, Acquisition, Examination and Analysis and Reporting. National Institute of Standards and Technology (NIST) also recommends these phases. Preservation is a process of securely maintaining custody of property without altering or changing the content of data that reside on devices and removable media. Acquisition is the process of obtaining information from a mobile device and its associated media. In this process, the potential digital evidence is extracted from the sources such as the mobile device’s internal memory, SIM card memory, and SD memory, using acquisition methods. Examination & Analysis are the processes used to uncover digital evidence such as hidden data. The results are obtained through applying established scientifically-based methods and should describe the content and state of the data fully. Finally, Reporting is the process of preparing a detailed summary of all the steps taken and conclusions reached in the investigation of a case.

### 3.2 Model collection and classification

This step includes collecting several MF models from a variety of sources, including books, journal papers, conference papers and reports that were found from Google Scholar, ScienceDirect, IEEE Xplore, PLOS One, Springer Link and Google. The collection of models was conducted using different keywords such as ‘‘mobile forensics model”, ‘‘smartphone forensics analysis”, ‘‘mobile forensics preservation”, ‘‘mobile forensics acquisition”, ‘‘mobile forensics examination”, ‘‘mobile forensics identification” and ‘‘evidence extraction of mobile device”. Among these collected models, some models cover all four phases of MF, while others cover three, two or even only one phase. Hence, based on the number of phases included, the model can be called either a “general model” or a “specific model”. The model is called “general model” if it can cover at least three phases of MF, otherwise the model is called “specific model”.

For this study, a total of 41 models were collected, from which 31 models were considered as general models and 10 models as specific models. These models were selected based on their clarity and how well domain knowledge is presented through the models. The collected models were then classified into three different sets (Set1, Set V1 and Set V2) for development and validation of the MFM. These sets are formed according to how broadly the models cover the four phases of MF. Set I, which includes 21 general models is used to create the initial metamodel, while Set V1 which includes 10 specific models and Set V2 which includes 10 general models are used for validation of the metamodel in Step 8.

The purpose of this first validation (Set V1) is to identify any missing concepts in the initial metamodel, because the specific models provide more information for each phase of the MF domain than provided by general models. While Set V1 concentrates on validating the MFM against specific MF models, the second validation (Set V2) focuses on generic MF models. It is worth mentioning that including the general models with wider coverage in this set will provide better indication of the frequency of concepts across the models, which is necessary to evaluate the importance of individual concepts included in the MFM. Table 2 shows the models in each set.

**Table 2:** MF model collection and classification.

Model		Year published	Cover Phase			
			Preservation	Acquisition	Examination & Analysis	Reporting
**Set1 for Metamodel development**						
1	Developing Process for Mobile Device Forensics [43]	2009	X	X	X	X
2	Symbian smartphones forensic process model [44]	2009	X	X	X	X
3	Windows Mobile Forensic Process Model [45]	2007	X	X	X	X
4	Smartphone Forensic Investigation Process Model [46]	2012	X	X	X	X
5	Smart-Phone DEFSOP [47]	2011	X	X	X	X
6	Enhanced Mobile Forensic Process Model [48]	2013	X	X	X	X
7	Framework for iPhone Forensic [49]	2011	X	X	X	X
8	Mobile Forensics using the Harmonised Digital Forensic Investigation Process [50]	2014	X	X	X	X
9	A quantitative approach to Triaging in Mobile Forensics [51]	2011	X	X	X	X
10	A Theoretical Process Model for Smartphones [52]	2013	X	X	X	X
11	Mobile Smart Device Investigation Process [53]	2015	X	X	X	X
12	Conceptual Evidence Collection and Analysis Methodology for Android Devices [54]	2015	X	X	X	X
13	Mobile Forensic Investigation Life Cycle Process [55]	2016	X	X	X	X
14	An Android Social App Forensics Adversary Model [56]	2016	X	X	X	X
15	Android cache taxonomy and forensic process [57]	2015	X	X	X	X
16	Thumbnail forensic recovery process for Android devices [58]	2015	X	X	X	X
17	Integrated Digital Forensic Investigation Model for smartphone [59]	2016	X	X	X	X
18	Framework of Digital Forensics for the Samsung Star Series Phone [60]	2011	X	X	X	X
19	Guidelines on Mobile Device Forensics [42]	2013	X	X	X	X
20	Mobile Forensics Model [61]	2016	X	X	X	X
21	An Approach for Mobile Forensics Analysis [62]	2015	X	X	X	X
**Set V1 for first validation**						
1	Digital evidence extraction and documentation from mobile devices [63]	2013		X		
2	ANDROPHSY–Forensic Framework for Android [64]	2015	X		X	X
3	Mobile Forensic Adversary Model [65]	2015			X	
4	A Mobile Forensics Model Based on Social Relations [66]	2014			X	
5	Evidence Data Collection through iPhone Forensic [67]	2012	X	X		
6	A General Collection Methodology for Android Devices [68]	2013		X	X	
7	Forensic analysis and security assessment of Android m-banking apps [69]	2016		X	X	
8	Logical acquisition and analysis of data from android mobile devices [70]	2015		X	X	
9	Smartphone Forensics: A Proactive Investigation Scheme [71]	2011	X	X		
10	CDCD-5 an Improved Mobile Forensics Model [72]	2012	X		X	
**Set V2 for second validation**						
1	Testing the Harmonised Digital Forensic Investigation Process Model-Using an Android Mobile Phone [73]	2013	X	X	X	X
2	Advances of Mobile Forensic Procedures in Firefox OS [74]	2014	X	X	X	
3	Acquisition and Analysis of Digital Evidence in Android Smartphones [75]	2011	X	X	X	
4	Generic Process Model for Smartphones Live Memory Forensics [76]	2014	X	X	X	X
5	Digital Forensics Process of Smartphone Devices [77]	2011	X	X	X	X
6	Guidelines on Cell Phone Forensics [5]	2007	X	X	X	X
7	Smart Handheld Device Digital Evidence Forensic Procedures [78]	2013	X	X	X	
8	Systematic Digital Forensic Investigation Model [79]	2011	X	X	X	X
9	The Forensic Process Analysis of Mobile Device [80]	2015		X	X	X
10	A Unified Forensic Investigation Framework of Smartphones [81]	2013	X		X	X

### 3.3 Concept extraction

This step is an important process in the metamodeling approach. The purpose of this process is to identify concepts among the models that could potentially be included in the MFM. Extracting concepts should be performed from the textual contents (main body) of a mobile forensic model in order to avoid any missing or unrelated concepts during extraction process. The main body contains the developed model. For instance, Xian’s model “Symbian smartphones forensic process model” [31] covered a five processes for Symbian smartphones. We extracted the related concepts under each of these processes. The extracted concepts should be related to the MF domain, otherwise, they were excluded. However, similarly to the procedures in [39, 41, 82, 83], the concepts were extracted manually from each model in Set I. We adapted the concept extraction process from [84–87]. A description of the concept extraction process is presented below:

- Concept Recognition: this step is based on a linguistic approach. The concept should contain noun or adjective + noun or compound noun to recognize it. For instance, “*Investigator* and *Court*” are a noun; “*Faraday Bag*, *Chain of Custody*” are compound noun, whereas “*Acquired Data*, *Volatile Evidence*” are adjective + noun.
- Concept categories: candidate concepts of the metamodel are represented as:
  - Actor (active concepts) such as (*Investigator*, *Forensic Specialist*, *Audience*).
  - Object (passive concepts) such as (*Evidence*, *Source*, *Result*).
  - Process (activities) such as (*Verification*, *Extraction*, *Documentation*).

**Fig 3:**
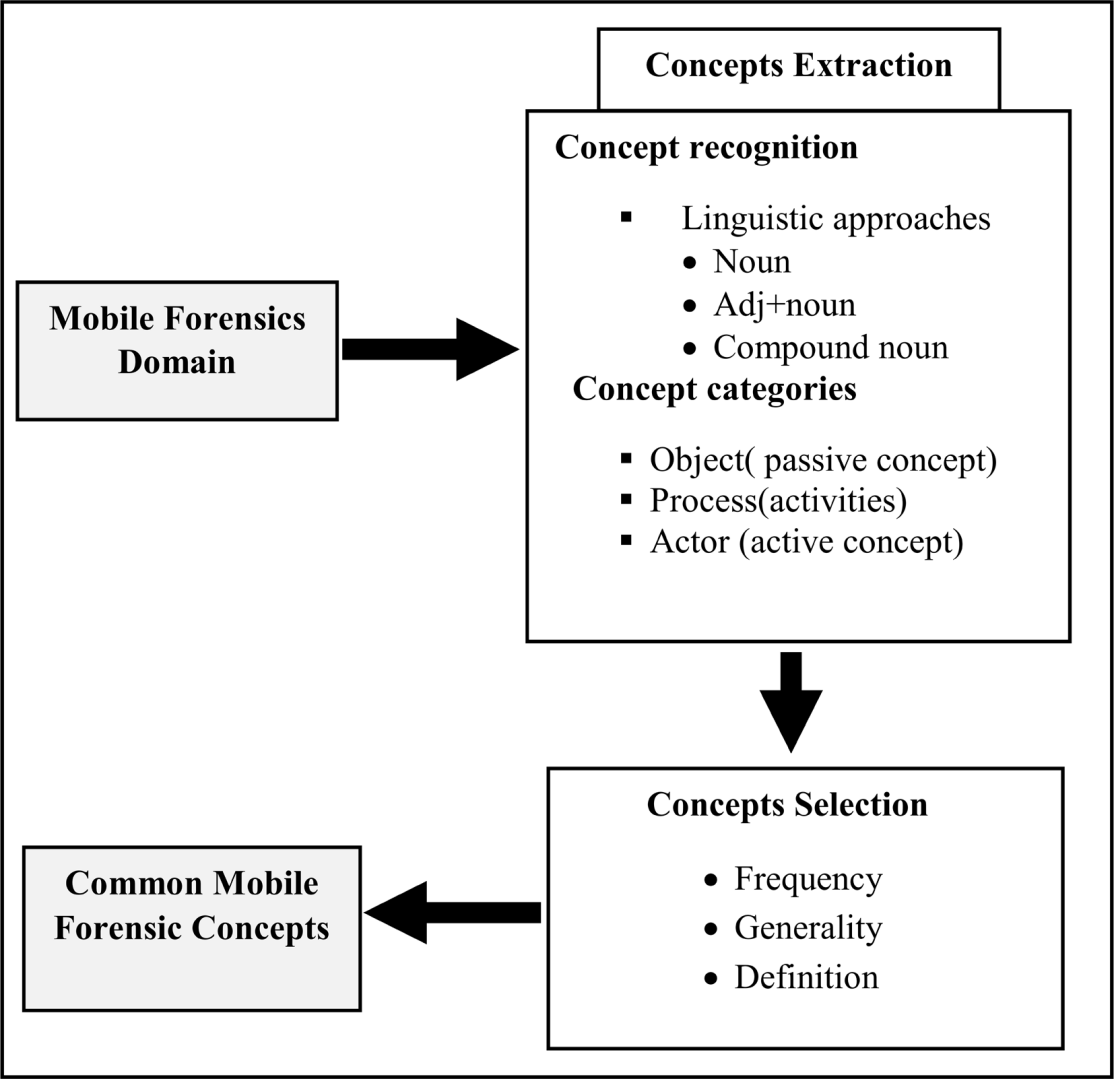
Concept extraction process.

The concept extraction process is shown in Fig 3. The outcomes of the concept extraction are shown in Table 3. We extracted 725 general concepts from Set 1 (including 21 models in total).

**Table 3:** Concept extraction.

Model	Concept	Total
Developing Process for Mobile Device Forensics [43]	Procedure, Chain of Custody, Information, Incident, Identification, Legal Authority, Search Warrant, Removable Data Storage, Mobile Device, Source, Potential Evidence, Forensic Tool, Documentation, Preparation, Drivers, Isolation, Faraday Bag, Radio Frequency Shielding, Extraction, Physical Memory Dump, logical Acquisition, Manual Extraction, Flash Memory Chip, Examination Data, Analyst, Examiner, File System, Verification, Hash Value, Integrity, Presentation, Prosecutor, Court, Investigator, Audience, Evidence, Jury, Archiving, Finding, Experience, Photographing, Backup, Equipment, Physical Acquisition, Unlocking Bootloader, Airplane Mode, Network Provider	47
Symbian smartphones forensic process model [44]	Preparation, Identification, Initial Information, Mobile Device, Forensic Tool, Policy, Analysis Data, Integrity, Pattern matching, Examination Data, Interpretation, Presentation, Review, Result, Evidence, Removable Media	16
Windows Mobile Forensic Process Model [45]	Preparation, Recording, Photographing, Sketching, Crime, Crime Scene, Investigator, Evidence Source, Assessment Crime, Authorization, Search Warrant, Experience, Mobile Device, PackagingAndSealing, Transportation and Storage, Jurisdictional Law, Chain of Custody, Integrity, People, External Storage Media, Survey, Recognition, Potential Evidence, Search Plan, Securing Scene, Environmental Circumstance, Shock, Humidity, Temperature, Victims, Suspect, Witness, Forensic Specialist, KeywordSearch, Documentation, Communication Shielding, Evidence Collection, Volatile Evidence, Non-Volatile Evidence, Forensic Tool, Instigation Procedure, Examination Data, Data Filtering, Validation, Pattern Matching, Tampering, Hashing Technique, Recovering Data, Analysis Data, Investigative Team, Reconstructing Event, Timeframe Analysis, Hidden Data Analysis, Application and File Analysis, Interpretation, Presentation, Results, Audience, Law Enforcement, Technical Expert, Legal Expert, Corporate Management, Court of Law, Conclusion, Evidence, Jury, Police Investigation, Review, Legal Constraint, Investigation strategy, Backup, Equipment, Source, Unlocking Bootloader	76
Smartphone Forensic Investigation Process Model [46]	Tool, Crime Scene, Search Warrant, Knowledge, Mobile Device, PackagingAndSealing, Transportation and Storage, Investigation Procedure, Legal Constraint, Legal Jurisdictional, Suspect, Authorization, Integrity, Investigator, Chain of Custody, Recording, Photographing, KeywordSearch, Crime-scene Mapping, Documentation, Tampering, Victim, Witness, Communication Shielding, Environmental Effect, Shock, Humidity, Temperature, Volatile Evidence, Non-volatile Evidence, External Storage, Cell Site Analysis, Law Enforcement, Examination Data, Data Filtering, Validation, Pattern Matching, Recovering Data, Forensic Specialist, Hashing Technique, Analysis Data, Reconstructing Event, Timeframe Analysis, Hidden Data Analysis, Application and File Analysis, Interpretation, Presentation, Audience, Technical Expert, Legal Expert, Jury, Corporate Management, Court of Law, Police Investigation, Conclusion, Review, Result, Systematic Strategy, Forensic Laboratory, Securing Scene, Airplane Mode, Cell Site Analysis, Local Service Provider	66
Smart-Phone DEFSOP [47]	Legislation, Documentation, Crime, People, Preparation, Mobile Device, Investigator, Searching Place, Forensic Tool, Integrity, Collecting information, Detaining Evidence, Analysis Data, Mobile Calendar, Call History, Message, Voicemail, Memory Card, Acquired Data, Crime Scene, Court, Result, Copy of Evidence, Judge, Equipment Identification, Presentation, Laboratory	27
Enhanced Mobile Forensic Process Model [48]	Preparation, Authorization, Search Warrant, Recording, Photographing, Sketching, Planning, Tool, Securing Scene, Survey, Recognition, Forensic Specialist, Device Mode, PackagingAndSealing, Transportation and Storage, Signal Isolation, Acquired Data, Hand-held device, Evidence, Laboratory Evidence, Volatile Evidence, Investigative Team, Examination Data, Analysis Data, Evidence, Backup, Hidden Data, Reconstructing Event, Presentation, Chain of Custody, Review, Audience, Result, Law Enforcement, Corporate Management, Legal Expert, Court Ruling, Crime, Seizure, Forensic Examiner	41
Framework for iPhone Forensic [49]	Tool, Forensic Investigator, Data Integrity, Logical Acquisition, Physical Acquisition, Suspect Device, Data Analysis, Text Evidence, Network Evidence, Audio-Visual Evidence, Online Activity Evidence, User Activity Evidence, Software, Backup, Retrieved Evidence, Evidence, Authority, Crime Scene, Cellular Provider	19
Mobile Forensics using the Harmonised Digital Forensic Investigation Process [50]	Investigation Procedures, Incident, Identification, First Responder, Investigator, Planning, Techniques, Preparation Equipment, Documentation, Incident Scene, Chain of Custody, Extraction, Evidence, Authorization, Investigative Team, Photographing, Recording Scene, Potential Evidence, Integrity, Transportation and Storage, Shock, Acquired Data, Logical Acquisition, Physical Acquisition, Analysis Data, Reconstructing Scene, Recovery, Evidence, Interpretation, Expert witness’s testimony, Presentation, Timestamp, Stakeholders, jury, Accused, Lawyers, Prosecutor, Validity, Investigation Conclusion, Decision, Laboratory, Retrieved Data, Internal Memory	45
A quantitative approach to Triaging in Mobile Forensics [51]	Device Identification, Crime Scene, Extraction, Data Triaging, Technique, Analysis Data, Evidence, Forensics Lab, Extracted Data, Investigator, Mobile Content, Mobile Phone	13
A Theoretical Process Model for Smartphones [52]	Transportation and Storage, Device, Isolation, Investigator, Faraday Bag, Documentation, Classification, Case, Forensic Tool, Suspect, Victim, Collecting Facts, Information Device, Forensic Examiner, Potential Evidence, Backup, Examination Data, Investigation Procedures, Analysis Data, Extracting Data, Evidence, Hashing Method, Verification, Internal Components, Removable Component, Interpretation, Presentation, Result, Stakeholder, Law Enforcement, Source	31
Mobile Smart Device Investigation Process [53]	Incident Detection, Crime Scene, Preparation, Sketching, Photographing, Recording, Chain of Custody, Target Device, First Responder, Assessment Incident, Investigation Plan, Potential Evidence, People, Forensic Personnel, Investigation Strategy, Identification, Isolating, Pattern Matching, Search Warrant, Documentation, Device Power, Recovering Data, Acquisition Method, Manual Acquisition, Logical Acquisition, Physical Acquisition, Integrity, Duplicate Evidence, Examination Data, Search, Filtering, Hidden Data, Visibility, Traceability, Validating, Evidence, Tool, External Evidence, Analysis Data, Reconstructing Event, Conclusion, Legal Expert, Investigator, Presentation, Summarizing, Court, Physical Evidence, Response Strategy, Acquired Data, Source, Rooting	53
Conceptual Evidence Collection and Analysis Methodology for Android Devices [54]	Procedure, Practitioner, Device, Faraday Bag, Photographing, Seizure, Practice, Disable Device Radio, Internal Memory, Physical Evidence, Filtering, Physical Collection, Device State, Potential Evidence, Forensic Procedure, Extraction, Suspect, Non-volatile Evidence, Integrity, Flash Memory, Forensic Tool, Organization, Hashing Algorithm, External Storage, Analysis Technique, Examination Data, Analysis Data, Evidence, KeywordSearch, Verification, Presentation, Finding, Court, Backup, Unlocking Bootloader, Airplane Mode, Rooting	39
Mobile Forensic Investigation Life Cycle Process [55]	Seizure, Identification, Planning, Preparation, Disable Network, Acquiring Mobile, Faraday Bag, Internal Memory, External Memory, Transportation and Storage, Laboratory, Crime, Storage Media, Chain of Custody, Data Analysis, Examination Forensic, Presentation, Legal Authority, Capturing, KeywordSearch, Source	21
An Android Social App Forensics Adversary Model [56]	Logical Forensic, Physical Forensic, Forensic Analysis, Tool, Examination, Evidence, Investigator, Findings, Android Phone, Internal Device Memory, Personal Information, Rooting	12
Android cache taxonomy and forensic process [57]	Law Enforcement, Forensic Examination, Classification, Forensic Practitioner, Practice, Forensic Analysis, Internal Storage, Mobile Device, Presentation, Court, Extraction, External Storage, Rooting	13
Thumbnail forensic recovery process for Android devices [58]	Identification, Mobile Device, Potential Evidence, Flash Memory, Tampering, Evidence, Physical Acquisition, Logical Acquisition, Manual Acquisition, Data Recovery, Extraction, Analysis Data, Hashing, Integrity, Matching, Presentation, Source, Unlocking Bootloader	19
Integrated Digital Forensic Investigation Framework for smartphone [59]	Preparation, Notification, Authorization, Seized Device, Incident Response, Securing Scene, Documentation, Crime, Scene, Event Triggering, Transportation and Storage, Communication Shielding, Volatile Evidence, Non-Volatile Evidence, Examination Data, Analysis Data, Reconstruction, Hashing, Presentation, Conclusion, Dissemination, Decision, Investigator	24
Framework of Digital Forensics for the Samsung Star Series Phone [60]	Preparation, Authorization, Forensic Examination, Transportation and Storage, Practice, Search, Seizure, Warrant, Witness, Evidence, Authority, First Responder, Crime Scene, Investigator, Equipment, Investigation Procedure, Disable Signal, Phone State, Live Acquisition, Manual Acquisition, Logical Acquisition, Capturing, Analysis Data, Presentation, Collected Data	25
Guidelines on Mobile Device Forensics [42]	Mobile Device, Identification, Securing Scene, Evaluating Scene, Potential Digital Evidence, Procedure, Seizure Device, Integrity, Preparing, Search, Documentation, Recording, Photographing, Evidence Collection, Memory Volatility, PackagingAndSealing, Transporting and Storing Evidence, Isolation, Faraday Cage, Decision, Filtering, Law Enforcement, Validation, Hidden Data Analysis, Equipment, Removable Media, Verification, Interviewing, Internal Memory, Forensic Examiner, Capturing, Forensic Specialist, Forensic Laboratory, Acquisition Method, Logical Acquisition, Physical Acquisition, Manual Extraction, Extraction, Recovering, Search Warrant, Forensic Tool, Examination Data, Copy of Evidence, Forensic Analyst, Potential Evidence, Suspect, Analysis Data, Hash Value, Application and File Analysis, Timeframe Analysis, Court of Law, Results, Evidence, Jurisdiction, Scene, Conclusion, Acquired Data, KeywordSearch, Source, Airplane Mode, Cell Site Analysis, Network Provider	62
Mobile Forensics Model [61]	Preparation, People, Investigation Team, First Responder, Securing Scene, Crime scene, Systematic Strategy, Legal Constraint, Evidence, Chain of Custody, Integrity, Cut Network Communication, Acquisition Method, Manual Acquisition, Logical Acquisition, Physical Acquisition, Mobile Device, Internal Memory, Non-Volatile Evidence, Volatile Evidence, Documentation, Legal Authority, Photographing, Examiner, Investigation, Transportation and Storage, Procedure, Humidity, Temperature, Environmental Effect, Forensics Lab, Examination Data, Collected Evidence, Copy of Evidence, Data Filtering, Validation, Detecting, Recovering Data, Forensics Tool, Analysis Data, Time frame Analysis, Presentation, Court of Law, Decision, Crime, Culprit, Evidence, Review, investigator, Result	52
An Approach for Mobile Forensics Analysis [62]	Investigator, Seizure, Wireless Network Off, Faraday Cage, Suspect, Crime Scene, Documentation, Forensic Lab, Tool, Forensic Analyst, Analysis, Forensic Analysis, External Memory, Forensic Examiner, Hash Function, Integrity, Presentation, Result, Audience, Collected Data, Evidence, Internal Memory, Source, Airplane Mode	24

### 3.4 Selection and identification of common concepts

In this step, we identified the common concepts from step 3 (containing 725 concepts in total) based on concepts that have been widely used in the domain of MF. However, some concepts used different name but with similar meaning. For example, the concepts "*Incident*" in models [43, 50], "*Case*" in model [52] and "*Crime*" in models [45, 47, 48, 55, 59, 61] have similar meaning. Hence, we grouped these concepts into one common concept: “*Crime*”, as shown in Table 4.In addition, the concepts that have a single name such as “*Securing Scene*” in models [42, 45, 46, 48, 59, 61] are considered as common concept. The remainder of selection of common concepts are shown in S1 Table. For the concepts that shared same meaning, we used the following features: Frequency (occurrence), Generality and Definition to select the name of the common concepts from them. Therefore, if two or more concepts have similar meanings, then the concept name with higher frequency, generality and definition will be selected for inclusion in the metamodel while the other names will be excluded. For example, the shared meaning of the concepts: *Classification*, *Identification* and *Recognition* is: ‘‘used by investigator to identify type of mobile device and its operating system, people in the crime scene, external data storage and potential evidence sources”. The concept ‘‘*Identification*” is selected as a common concept because it has higher frequency in more models than *Classification* and *Recognition*. Hence ‘‘*Identification*” is included in the metamodel and *Classification* and *Recognition* are excluded. Indeed, the main priority for selecting the common concept is the high frequency (occurrence) of the concept among all models. The outcome of this step is selecting 82 common concepts, as shown in S1 Table.

**Table 4:** A sample of selection of common concepts.

No	Common Concept	Concepts	Frequency	Generality	Definition
1	Chain of Custody	Chain of Custody	9	1	1
2	Crime	Incident	2	1	1
		Case	1	0	0
		Crime	6	1	1
3	Securing Scene	Securing Scene	6	1	1
4	Identification	Identification	8	1	1
		Recognition	2	1	1
		Classification	2	1	1

*Legend*: (**Frequency**) *=* number of occurrence of a concept among models; (**Generality**) *=* **1** if the concept is a general, otherwise = ‘**0**’; *(***Definition***) =* ‘**1**’ if the concept has a definition, otherwise = ‘**0**’.

### 3.5 Short-listing and reconciliation of definitions

In this step, we provide a short list of definitions for every selected concept in step 4. A harmonization of the definitions in the metamodel is required, when two or more concepts have the same definition, or even two or more concepts have the same concept name. The chosen definition for each concept must be a precise definition and widely agreed in the mobile forensic domain [39, 82].

In addition, differences between definitions were reconciled to ensure an internally consistent set of metamodel terms. Definitions were chosen based on consistency with earlier choices, where possible; otherwise, hybrid definitions created from multiple sources were introduced. If there is a different use of concept definition between two or more sources, the process was to select the usage that was most coherent with the rest of the set of chosen concepts trying at all times to preserve generality. For instance, the concept of “*Documentation*” was defined differently in four models: Kaur [62] defines it as “Document all the steps”. Ayers [42] defines it as “an essential activity in providing individuals the ability to re-create the process from beginning to end and documenting the crime Scene (Photographing, Sketching, and Recording). Dancer [52] defines it as “an activity that takes place within each phase of forensics investigation and therefore should not be a standalone activity in any forensics examination”. Mumba [50] defines it as “a process to improve efficiency by ensuring documentation of all steps is clearly undertaken during a mobile forensic investigation”. Ramabhadran [45] defines it as “a continuous activity required in all the stages and is quite critical for maintaining proper chain of custody”. As a result, the concept of “*Documentation*” in our metamodel is defined as “*a continuous activity required in all the phases of mobile forensic and used for documenting the crime scene through (Photographing*, *Sketching and Recording*)”. A sample of the list of short definitions is shown in Table 5. The rest of the concept definitions are shown in S2 Table.

**Table 5:** Sample of concept definitions.

Concept	Definition
Chain of Custody	A process that tracks the movement of evidence through its collection, preservation, and analysis lifecycle by documenting each person who handled the evidence, the date/time it was collected or transferred.
Documentation	A continuous activity required in all the stages and used for documenting the crime Scene (Photographing, Sketching, and Recording).
Extraction	A process to acquire data from mobile phone using acquisition methods which are manual acquisition, logical acquisition, and physical acquisition.
PhysicalAcquisition	A process to facilitate the examiner to search the contents of the removable media and potentially recover deleted files.
ForensicExaminer	Has ability to gather information about the individuals, determine the exact nature of the events that occurred, construct a timeline of events, uncover information that explains the motivation for the offense and discover what tools are used.

### 3.6 Classification of common concept

In this step, selected concepts are classified into one of the MF phases: *Preservation*, *Acquisition*, *Examination & Analysis and Reporting* [5, 42]. Classification into the phases is shown in Table 6.

**Table 6:** Classification of concepts.

MF Phase	Concepts
**preservation**	Crime; InvestigationProcedure; ChainOfCustody; LegalAuthority; SearchWarrant; MobileDevice; PotentialEvidence; Documentation; Preparation; Isolation; FaradayBag; Investigator; CrimeScene; Authorization; People; Packaging&Sealing; TransportingAndStorage; Identification; Planning; Shock; Humidity; Temperature; Victim; Suspect; Witness; Recording; Photographing; Sketching; InvestigationStrategy; SecuringScene; FirstResponder; Equipment; Collection; ForensicsLab; AirplaneMode; Source; Rooting; UnlockingBootloader; CellSiteAnalysis; NetworkProvider
**Acquisition**	Documentation; ChainOfCustody; PhysicalAcquisition; LogicalAcquisition; ManualAcquisition; VolatileEvidence; Non-VolatileEvidence; AcquiredData; AcquisitionMethod; Imaging; InternalMemory; ExternalStorage; ForensicTool; Backup; Extraction; PotentialEvidence; MobileDevice; ForensicExaminer; Hashing; Integrity
**Examination and Analysis**	AcquiredData; Documentation; ChainOfCustody; Verification; Integrity; PatternMatching; ForensicSpecialist; DataFiltering; Validation; Tampering; RecoveringData; ReconstructingEvent; TimeframeAnalysis; HiddenDataAnalysis; ApplicationandFileAnalysis; ForensicsLab; ExaminedData; ForensicTool; Evidence AnalysisData; ExaminationData; KeywordSearch
**Reporting**	Documentation; ChainOfCustody; Presentation; CourtOfLaw; Audience; LawEnforcement; TechnicalExpert; LegalExpert; Jury; Conclusion; Interpretation; Review; Result; Decision; Evidence; Archiving; Investigator

### 3.7 Relationship identification among concepts

**Fig 4:**
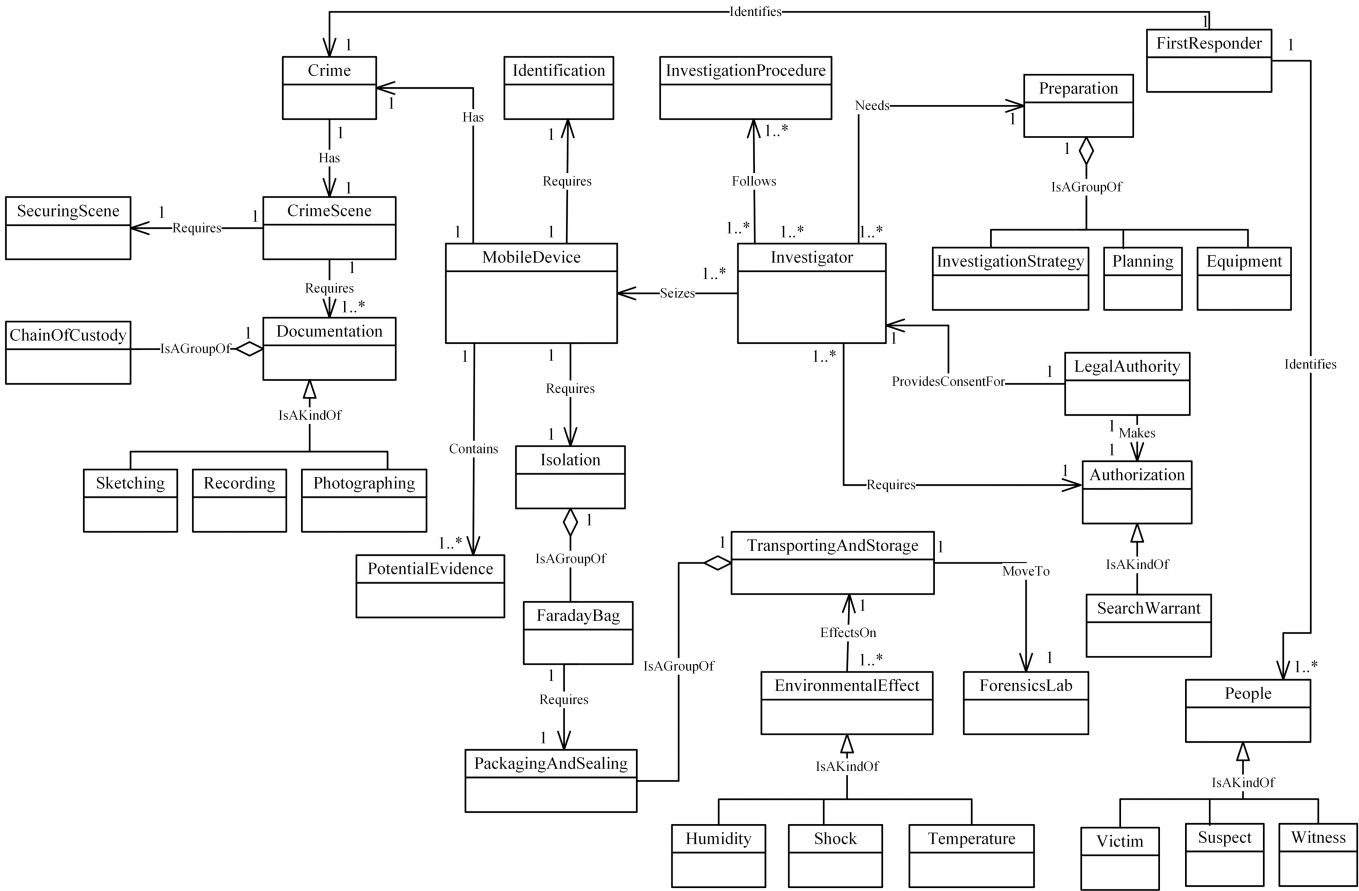
MFM 1.0: Preservation -phase class of concepts.

**Fig 5:**
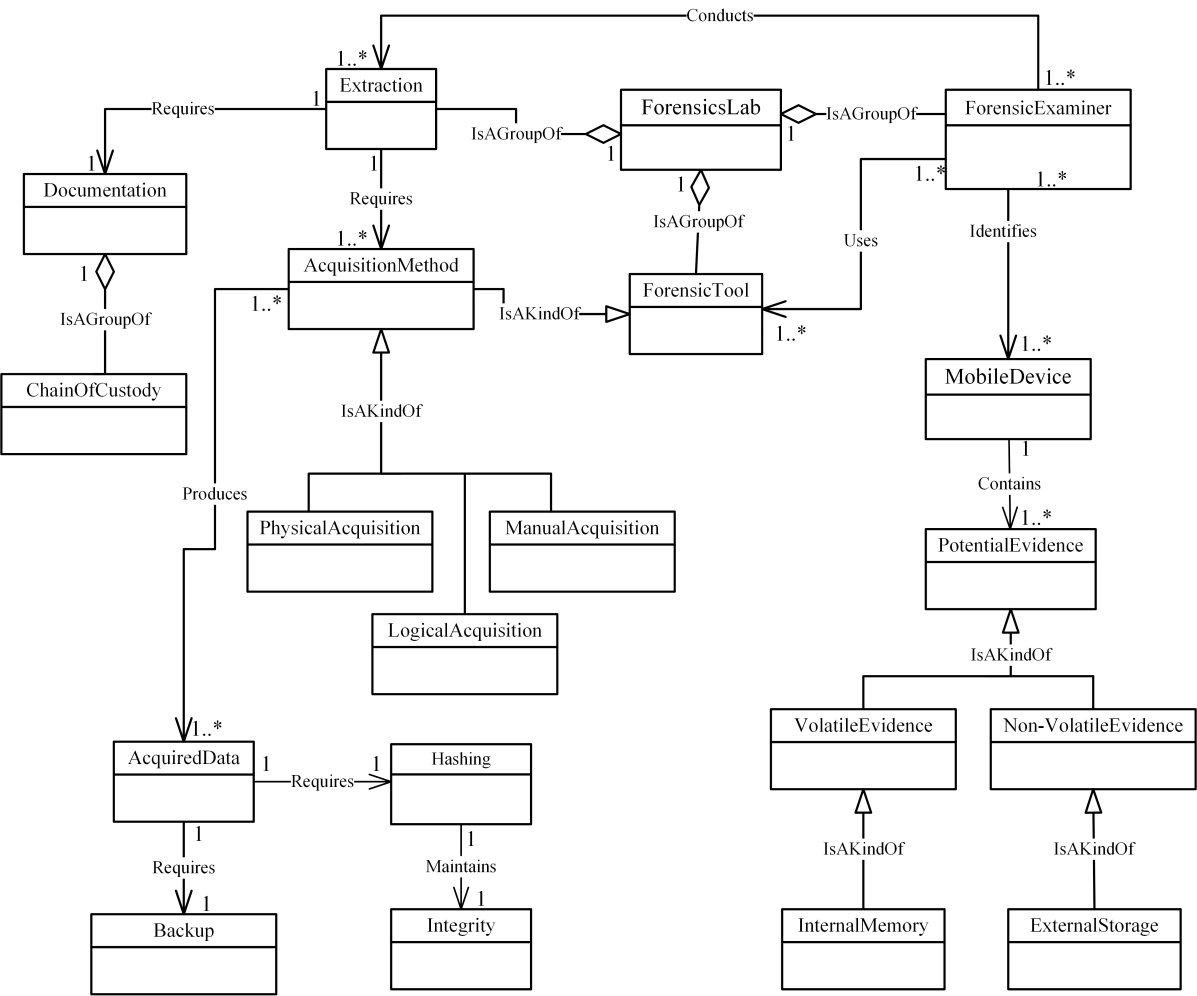
MFM 1.0: Acquisition -phase class of concepts.

**Fig 6:**
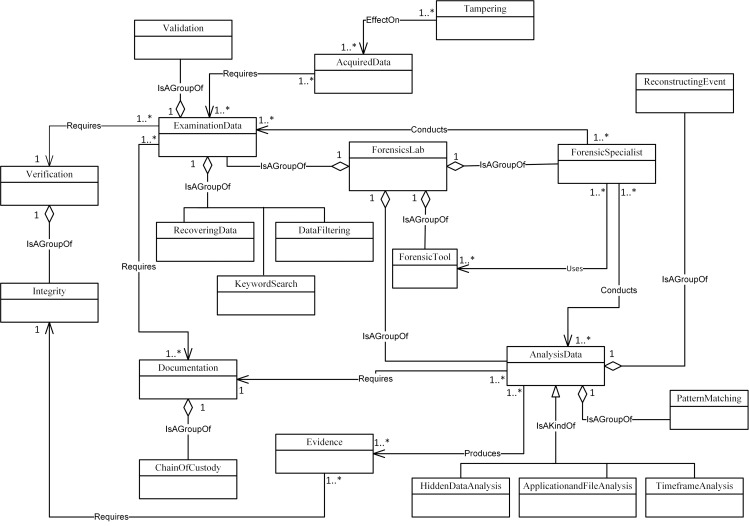
MFM 1.0: Examination & analysis -phase class of concepts.

**Fig 7:**
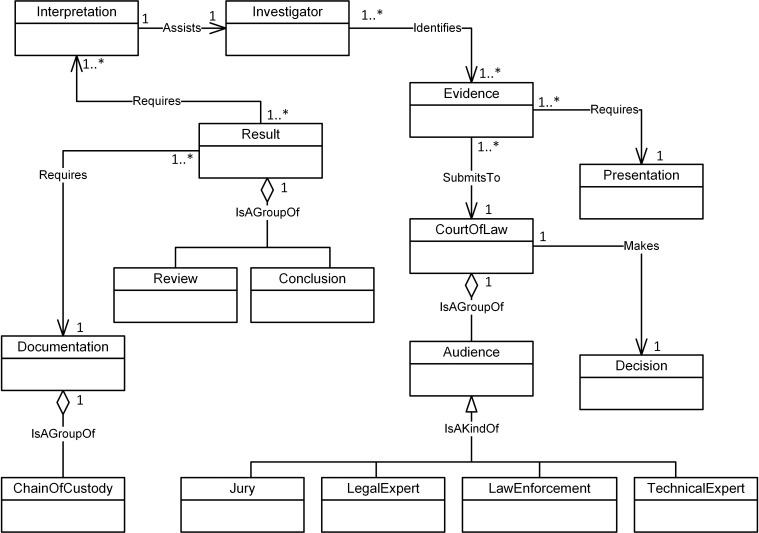
MFM 1.0: Reporting -phase class of concepts.

In this step, we determine the relationships between our MFM concepts. Mobile forensics investigation has four common phases, which are preservation, acquisition, examination and analysis and reporting in. Therefore, the resultant MFM is represented in four different diagrams which are: the Preservation-phase, the Acquisition-phase, the Examination and analysis-phase and the Report-phase. Figs 4–7 illustrate our initial MFM 1.0 diagrams for each phase. The resultant metamodel includes the relationships between concepts and represents the semantics of the MF domain. Therefore, we established the relationships between concepts, based on the semantic language, which were discovered and identified during survey of MF models. We used three symbols of relationships which are *Association*; *Specialization*; and *Aggregation*. Association indicates functional relationships between concepts. Specialization represents hierarchies between concepts using relationship ‘Is A Kind Of’. Aggregation represents relationships between concepts that are composed of other concepts using relationship ‘Is A Group Of’. For example, the Acquisition-phase class (Fig 5) has a central concept, *ForensicLab*. The aggregation symbol is used to describe relationships between *ForensicLab* concepts and other concepts including *Extraction*, *ForensicTool* and *ForensicExaminer*. Another example of relationship between concepts is the association. This describes relations between ‘*Evidence*’ and ‘*Presentation*’ concepts in the Reporting-phase class (Fig 7). The relationship between ‘*InternalMemory*’ and ‘*VolatileEvidence*’ concepts represents using ‘Is A Kind Of’ in the Acquisition-phase class (Fig 5).

MF is a continuous process with activities linking phases at different points. Correspondingly, in our MFM, relationships between concepts are identified not only among concepts within the same phase, but also among concepts from different phases. Concepts from classes in different phases can be linked and a continuous MF process can be formed. Linkages across phases are established either through relationships among concepts from different phases or through common concepts among phases. For example, an association relationship ‘*Requires*’ can link the concept of “*ForensicTool*” (from the Acquisition-phase) to the concept “*Preparation*” (from the Preservation phase). Another example of a relationship that links two concepts across two phases is an association relationship ‘*Requires*’ that is used to create a link between the concept “*Evidence*” in the Reporting -phase class and the “*Collection*” concept in the Preservation -phase class. Table 7 illustrates examples of relationships that link concepts from different phases. Additionally, Linkages across phases are also established through common concepts between phases. The use of the concept “*Crime*” shows that the investigation task should start from the preservation phase in the mobile forensic investigation process, while the use of the concept “*Documentation*” shows that the four phases require overlapping sets of documentation for their phase activities.

**Table 7:** Examples of relationships among concepts in MFM.

Concept 1	Relationship	Concept 2	Metamodel Phase
Investigator	Association—‘follows	InvestigationProcedure	Preservation / see Fig 4
MobileDevice	Association—‘Requires’	Isolation	Preservation / see Fig 4
SearchWarrant	Specialisation—‘IsAKindOf’	Authorization	Preservation / see Fig 4
FaradayBag	Aggregation—‘isAGroupOf’	Isolation	Preservation / see Fig 4
Evidence	Association—‘Requires’	Presentation	Reporting/ see Fig 7
Audience	Aggregation—‘isAGroupOf’	CourtOfLaw	Reporting/ see Fig 7
ForensicSpecialist	Association—‘Conducts’	ExaminationData	Examination & Analysis/ see Fig 6
MobileDevice	Association—‘Contains	PotentialEvidence	Acquisition/ see Fig 5
ForensicTool	Aggregation—‘isAGroupOf’	ForensicsLab	Examination & Analysis/ see Fig 6
InternalMemory	Specialisation—‘IsAKindOf’	VolatileEvidence	Acquisition/ see Fig 5
ForensicTool	Association—‘Requires’	Preparation	Acquisition to Preservation (inter phases) see Figs 5 and 4
Evidence	Association—‘Requires’	Collection	Reporting to Preservation (inter phases) see Figs 7 and 4

### 3.8 Metamodel validation

In this section, we will discuss the validation process of our proposed MFM. The purpose of validation process is to measure the soundness and quality of proposed metamodel [88]. A metamodel requires validation to meet the requirements of generality, expressiveness and completeness of the artifact. In addition, to insure the completeness and correctness of the proposed metamodel, validation of the metamodel is required. For the validation process, the following two commonly used techniques [89, 90] were used:

1. ***Comparison with other Models*—**This technique is used to verify that each concept of a validation model can be represented with some of the metamodel concepts. In this technique, we added some concepts to the metamodel.
2. ***Frequency-based Selection—***The purpose of this validation technique is to verify the frequency of the metamodel concepts appearing in a set of models. In this technique, we deleted some concepts from the metamodel.

These validation techniques are described in the next subsections.

#### 3.8.1 Comparison with other models

**Fig 8:**
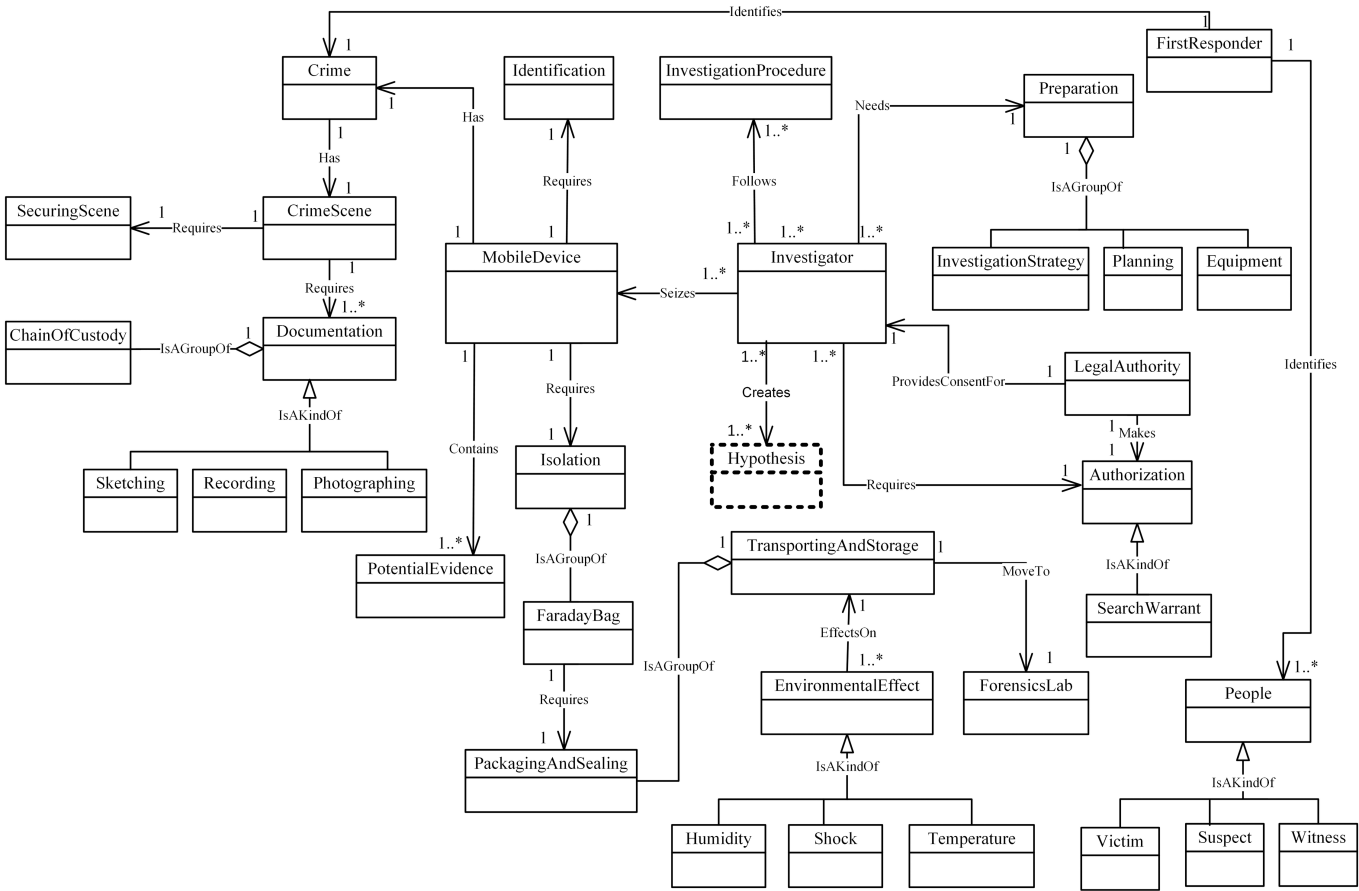
A validated version of preservation -phase class of concepts.

**Fig 9:**
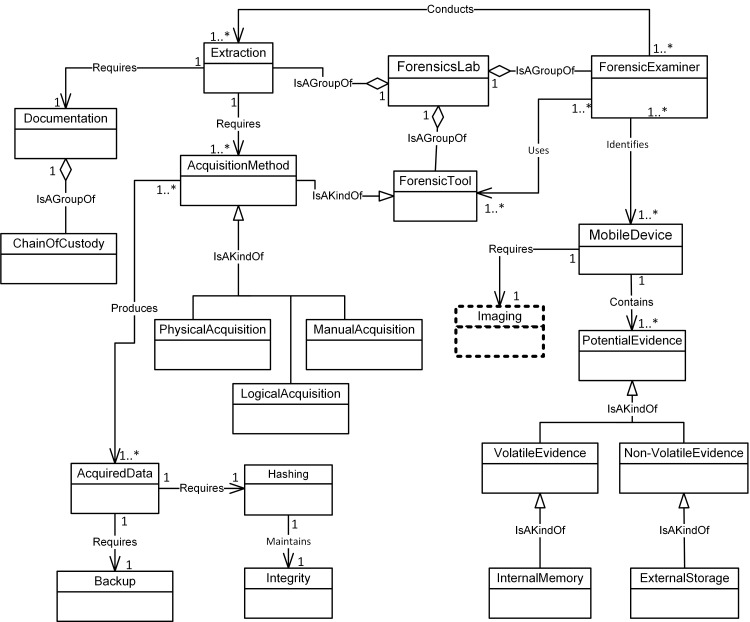
A validated version of acquisition -phase class of concepts.

**Fig 10:**
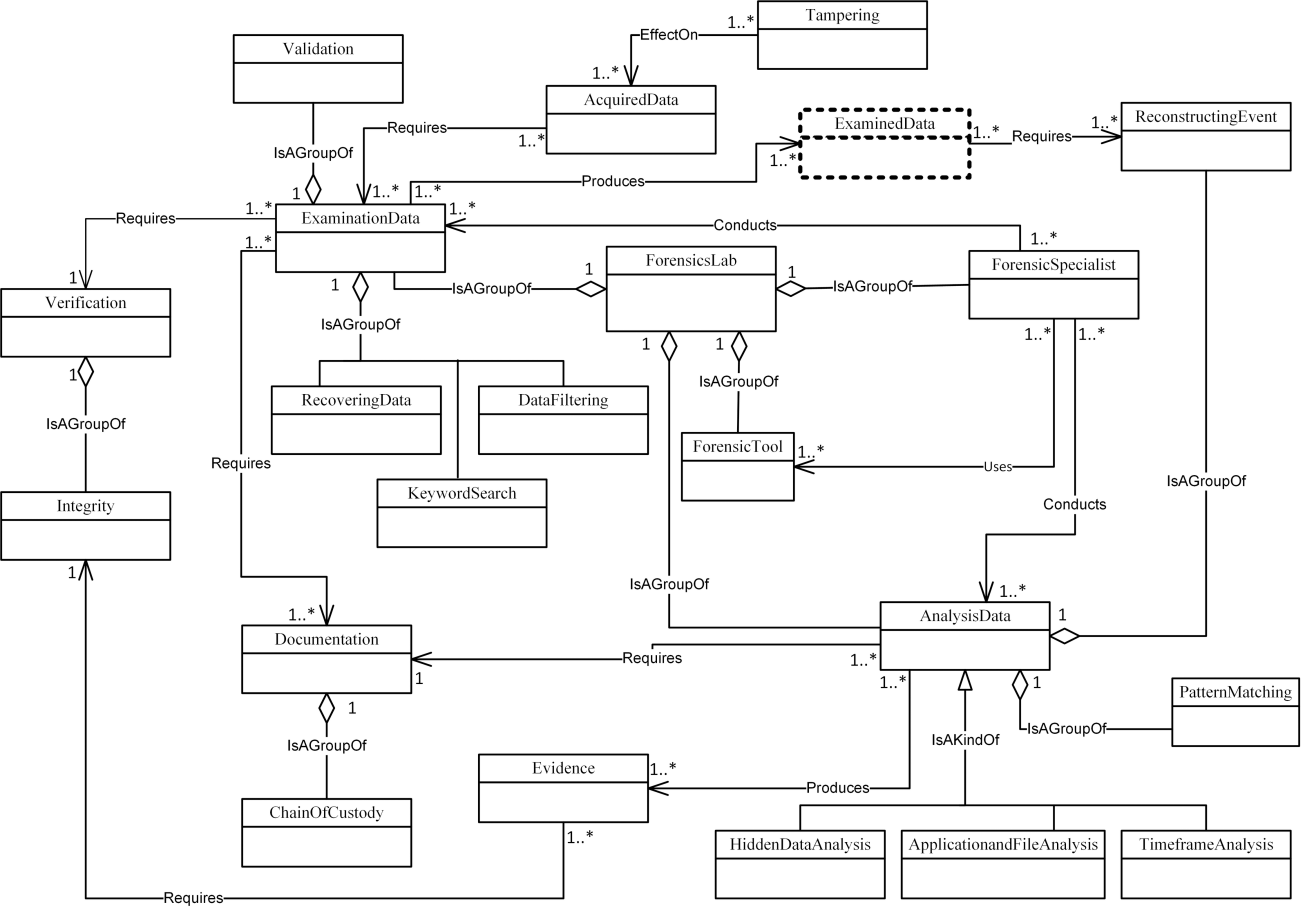
A validated version of examination & analysis -phase class of concepts.

**Fig 11:**
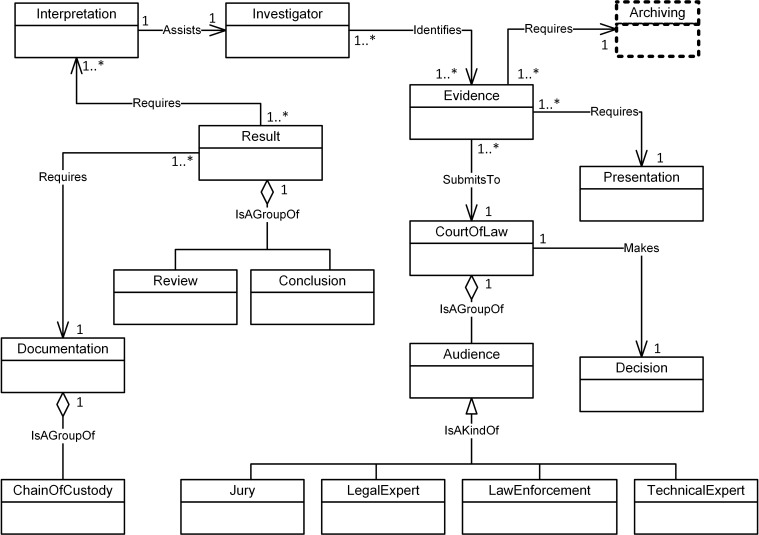
A validated version of reporting -phase class of concepts.

The purpose of this validation technique is to ensure that each model included in Set V1 is represented in MFM (shown in S3 Table). For example, if a concept of some model in Set V1 could not be represented in MFM, then we consider this concept as a candidate concept to add to MFM. In this process, we added four new concepts to MFM. Table 8 illustrates these new concepts. These four were added to MFM: Hypothesis, Imaging, DataExamined and Archiving as shown in Figs 8–11. The relationships between the new concepts and the concepts that comprise the MFM are shown in Table 9. The outcome of this technique was version MFM 1.1.

**Table 8:** Four new added concepts based on validation through comparison with10 models of Set V1.

Concept	MFM Phase	Definition
Hypothesis	Preservation	Gives an idea to the investigator what evidence must be collected and he can choose the appropriate tool according to type of mobile phone
Imaging	Acquisition	Use software to copy all electronic data on a device, performed in a manner that ensures the information is not altered
DataExamined	Examination & Analysis	Output of examination process
Archiving	Reporting	A necessary process to retain the data in a useable format for the ongoing court process, future reference, and for record keeping requirements

**Table 9:** List of relationships added to MFM.

Concept 1	Relationship	Concept 2	MFM Phase
Investigator	Association—‘Creates’	Hypothesis	Preservation
MobileDevice	Association—‘Requires’	Imaging	Acquisition
ExaminationData	Association—‘Produces’	DataExamined	Examination & Analysis
Evidence	Association—‘Requires’	Archiving	Reporting

#### 3.8.2 Frequency-based selection

**Fig 12:**
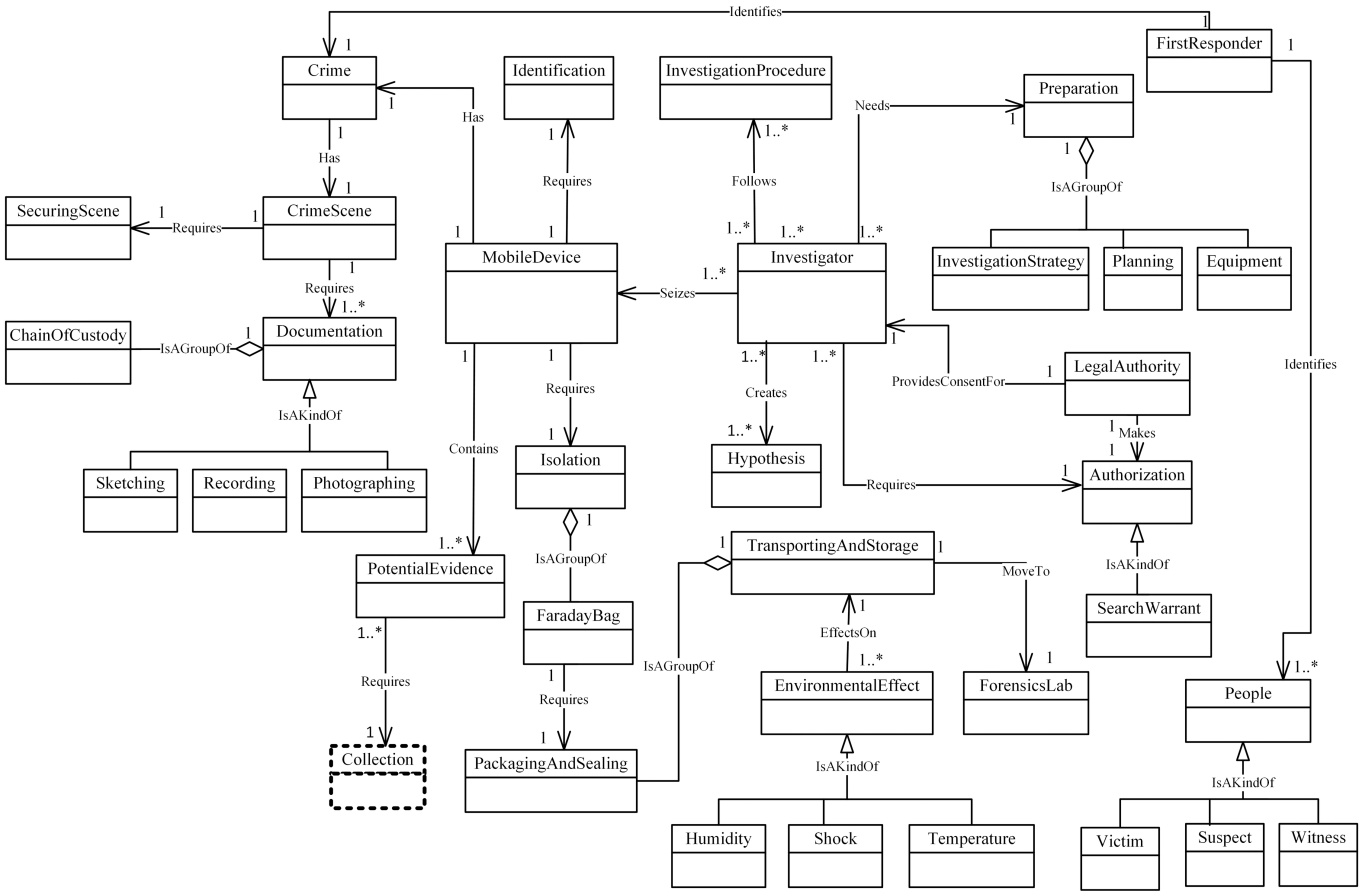
A validated version of preservation -phase class of concepts.

We used 10 models (Set V2 in Table 2) to perform this validation. The purpose of this technique is to evaluate the importance of individual concepts in the model developed [91]. This technique preforms two tasks. In the first task, we collect concepts from model Set V2 and compare them with concepts in the MFM 1.1, as shown in S4 Table. From this task, not all phases were changed to the same extent e.g.: the Preservation-phase of MFM 1.1 only gained the Collection concept as shown in Fig 12. The second task of frequency-based selection validation is to score each concept according to its frequency. Concepts which have a low score are revisited and are liable for deletion. To estimate an importance value for each concept in MFM, we used ‘Degree of Confidence (DoC)’. This value identifies the expected probability that a MFM concept is used in a randomly chosen mobile forensic model. Doc is defined as follows:

$$Degree\;of\;Confidence\;\left(Doc\right)=\frac{Frequency\;of\;Concept}{Total\;Model\;of\;Set\;V2}\times 100\%$$

The following five categories of concepts based on their DoC are defined:

1. Very Strong (DoC value: 100–70%).
2. Strong (69–50%).
3. Moderate (49–30%).
4. Mild (29–11%).
5. Very Mild (10–0%).

Very Strong refers to a concept that appears many times in Set V 2 models, while Very Mild is the other end of the scale. For example, the MFM concept *Identification* has a strong DoC value of 80%:

$$DoC\;\left(Identification\right)=\frac{8}{10}\times 100\%=80\%$$

Tables 10–13 have three main parts. Left part of tables contains concepts for each phase in the MFM1.1. The middle part of tables contains 10 models for Set V2 that were used to compare their concepts against concepts of MFM1.1. The right side of tables contains concept frequency (score) for each concept. Each row of these tables contains concepts for each phase in the MFM1.1.

**Table 10:** Frequency result of preservation-phase concepts.

MFM1.1 Preservation Concepts		Model Set V2										Concept Frequency
		1	2	3	4	5	6	7	8	9	10	
1	Crime	√			√	√	√	√	√	√		**7**
2	InvestigationProcedure	√	√	√			√	√	√			**6**
3	ChainOfCustody	√					√		√	√	√	**5**
4	LegalAuthority				√		√	√				**3**
5	SearchWarrant				√		√	√	√	√		**5**
6	MobileDevice	√	√	√	√	√	√	√		√	√	**9**
7	Source	√			√	√	√		√			**5**
8	PotentialEvidence	√				√	√		√			**4**
9	Documentation	√	√	√	√	√	√		√	√	√	**9**
10	Preparation	√	√		√			√	√		√	**6**
11	Isolation			√	√	√	√		√	√	√	**7**
12	FaradayBag	√				√	√		√			**4**
13	Investigator	√	√		√	√	√	√	√	√	√	**9**
14	CrimeScene	√			√	√	√	√	√		√	**7**
15	Authorization	√			√		√		√			**4**
16	People				√			√	√			**3**
17	PackagingAndSealing		√		√	√	√		√			**5**
18	TransportationAndStorage	√				√	√	√	√			**5**
19	Identification	√			√	√	√	√	√	√	√	**8**
20	Planning	√					√	√	√			**4**
21	Shock						√		√			**2**
22	Humidity						√	√	√			**3**
23	Temperature						√	√	√			**3**
24	Victim				√			√	√			**3**
25	Suspect			√	√	√	√	√	√			**6**
26	UnlockingBootloader			√	√		√					**3**
27	Witness				√		√		√			**3**
28	Recording	√					√	√	√			**4**
29	AirplaneMode			√			√					**2**
30	Photographing	√			√		√		√			**4**
31	Sketching				√				√			**2**
32	CellSiteAnalysis				√		√					**2**
33	InvestigationStrategy							√	√			**2**
34	NetworkProvider	√					√	√				**3**
35	SecuringScene				√		√		√		√	**4**
36	FirstResponder	√					√					**2**
37	Rooting	√		√	√							**4**
38	Equipment	√		√			√					**3**
39	ForensicLab				√	√	√	√		√		**5**
40	EnvironmentalEffect						√					**1**
41	Hypothesis	√			√					√		**3**

**Table 11:** Frequency result of acquisition -phase concepts.

**MFM1.1** **Acquisition Concepts**		**Model Set V2**										**Concept** **Frequency**
		**1**	**2**	**3**	**4**	**5**	**6**	**7**	**8**	**9**	**10**	
1	ChainOfCustody	√					√		√	√	√	**5**
2	Documentation	√	√	√	√	√	√	√	√		√	**9**
3	PhysicalAcquisition	√	√	√			√					**4**
4	LogicalAcquisition	√	√	√			√					**4**
5	ManualAcquisition			√			√					**2**
6	MobileDevice	√	√	√	√	√	√	√		√	√	**9**
7	VolatileEvidence				√				√		√	**3**
8	PotentialEvidence	√				√	√		√			**4**
9	Non-VolatileEvidence								√		√	**2**
10	AcquiredData				√	√	√					**3**
11	AcquisitionMethod			√			√				√	**3**
12	InternalMemory		√	√			√			√	√	**5**
13	ExternalStorage					√	√		√	√		**4**
14	Imaging		√	√		√	√			√		**5**
15	ForensicTool	√	√	√	√	√	√	√	√	√	√	**10**
16	Backup	√		√			√	√	√			**5**
17	ForensicExaminer			√			√			√		**3**
18	ForensicsLab				√	√	√	√		√		**5**
19	Extraction				√		√	√				**3**
20	Hashing		√		√	√				√		**4**
21	Integrity	√	√			√	√		√	√	√	**7**

**Table 12:** Frequency result of examination and analysis -phase concepts.

MFM1.1 Examination and Analysis Concepts		Model Set V2										Concept Frequency
		1	2	3	4	5	6	7	8	9	10	
1	AcquiredData				√	√	√					**3**
2	Documentation	√	√	√	√	√	√	√	√		√	**9**
3	ChainOfCustody	√					√		√	√	√	**5**
4	Evidence	√	√	√	√	√	√	√	√	√	√	**10**
5	Verification		√				√					**2**
6	Hashing			√		√	√			√		**4**
7	Integrity	√	√			√	√		√	√	√	**7**
8	PatternMatching								√			**1**
9	ForensicSpecialist			√		√	√		√			**4**
10	DataFiltering						√		√			**2**
11	Validation						√		√			**2**
12	RecoveringData			√	√		√			√		**4**
13	ReconstructingEvent	√							√			**2**
14	TimeframeAnalysis						√		√			**2**
15	HiddenDataAnalysis						√		√			**2**
16	AnalysisData	√	√	√	√	√	√	√	√	√	√	**10**
17	ExaminationData	√	√	√	√	√	√	√	√		√	**9**
18	ApplicationandFileAnalysis						√		√			**2**
19	ForensicsLab				√	√	√	√		√		**5**
20	ExaminedData	√									√	**2**
21	ForensicTool	√	√	√	√	√	√	√	√	√	√	**10**
22				√			√		√	√		**4**
23	Tampering											**0**

**Table 13:** Frequency result of reporting -phase concepts.

MFM1.1 Reporting Concepts		Model Set V2										Concept Frequency
		1	2	3	4	5	6	7	8	9	10	
1	Presentation	√			√	√			√	√	√	**6**
2	Documentation	√	√	√	√	√	√	√	√		√	**9**
3	ChainOfCustody	√					√		√	√	√	**5**
4	CourtOfLaw	√			√		√	√	√	√		**6**
5	Archiving				√		√					**2**
6	Audience	√				√			√	√		**4**
7	LawEnforcemen			√			√		√	√		**4**
8	TechnicalExpert								√			**1**
9	LegalExpert								√			**1**
10	Jury				√	√			√			**3**
11	Conclusion	√					√			√		**3**
12	Investigator	√	√		√	√	√	√	√	√	√	**9**
13	Interpretation	√			√		√					**3**
14	Review				√				√		√	**3**
15	Result		√		√	√		√	√	√		**6**
16	Decision	√			√							**2**
17	Evidence	√	√	√	√	√	√	√	√	√	√	**10**

In Tables 10–13, we compared each concept of the Preservation, Acquisition, Examination & Analysis and Reporting phases against the models of Set V2 to find concept frequency for each concept in these models. The results show that the concepts *EnvironmentalEffect*, *FirstResponder*, *InvestigationStrategy*, *Sketching* and *Shock*) in preservation-phase (Table 10) have low score, whereas concepts such as *Crime*, *MobileDevice*, *Documentation* and *Investigator* have a high score. In Table 11, the acquisition-phase has two concepts with low score which are *ManualAcquisition* and *Non-VolatileEvidence* concepts. The concepts *ForensicTool*, *Documentation* are examples of high score in this phase.

The concepts such as *Tampering*, *HiddenDataAnalysis*, *TimeframeAnalysis*, and *PatternMatching* have low score in the Examination & Analysis-phase in Table 12, whereas the concepts such as *AnalysisData*, *ExaminationData*, *ForensicTool* and *Documentation* have a higher score in this phase. In Table 13, the concepts *Evidence*, *Result*, *Investigator*, and *CourtOfLaw* are examples of concepts with high score, whereas concepts such as *Archiving*, *Conclusion* and *TechnicalExpert* have a low score in the Reporting-phase. The concepts with higher score mean these concepts are more important in the MF domain. In contrast, the concepts that have a low score are revisited and are liable for deletion.

The DoC classification of all MFM concepts is shown in Table 14: 12 concepts in MFM1.1 are categorized as ‘Very Strong’, 16 are ‘Strong’, 35 are ‘Moderate’, 17 are ‘Mild’ and 5 concepts are ‘Very Mild’. The five very mild concepts are *EnvironmentalEffect*, *PatternMatching*, *TechnicalExpert*, *LegalExpert* and *Tampering*. Including them in the MFM requires a reassessment. *Tampering* is deleted because the DoC value of this concept was 'zero', which means this concept is rarely recognized in the mobile forensic models. By revisiting MFM, it is found that the other four (*EnvironmentalEffect*, *PatternMatching*, *TechnicalExpert* and *LegalExpert*), are to be kept as they are common across varying MF domains.

**Table 14:** Degree of confidence of concepts for MFM after frequency-based selection.

DoC Classification	MFM Concepts
**100–70%** **(Very Strong)**	Crime, MobileDevice, Documentation, Isolation, Investigator, CrimeScene, ForensicTool, Integrity, AnalysisData, ExaminationData, Evidence, Identification
**69–50%** **(Strong)**	InvestigationProcedure, ChainOfCustody, SearchWarrant, Source, Preparation, PackagingAndSealing, TransportationAndStorage, Suspect, ForensicLab, InternalMemory, Imaging, Backup, ForensicsLab, Result, Presentation, CourtOfLaw,
**49–30%** **(Moderate)**	LegalAuthority, PotentialEvidence, FaradayBag, Authorization, People, Planning, Humidity, Temperature, Victim, Witness, Recording, Photographing, SecuringScene, Equipment, Hypothesis, PhysicalAcquisition, LogicalAcquisition, VolatileEvidence, AcquiredData, AcquisitionMethod, ExternalStorage, ForensicExaminer, NetworkProvider, Extraction, Hashing, UnlockingBootloader, ForensicSpecialist, Rooting, RecoveringData, Audience, LawEnforcemen, Jury, Conclusion, Interpretation, Review,
**29–11%** **(Mild)**	Shock, Sketching, InvestigationStrategy, FirstResponder, ManualAcquisition, Non-VolatileEvidence, Verification, HiddenDataAnalysis, Validation, ReconstructingEvent, TimeframeAnalysis, ApplicationandFileAnalysis, ExaminedData, Decision, AirplaneMode, Archiving, KeywordSearch, CellSiteAnalysis
**10–0%** **(Very Mild)**	EnvironmentalEffect (√), PatternMatching (√), TechnicalExpert (√), LegalExpert (√), Tampering **(x)**
	**(√) =** Keep the concept, (X) = Delete the concept

**Fig 13:**
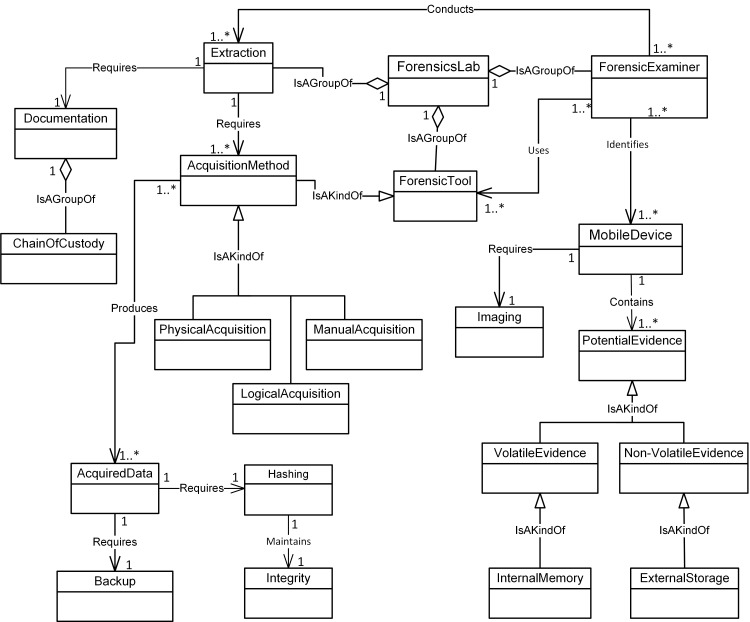
A validated version of acquisition -phase class of concepts.

**Fig 14:**
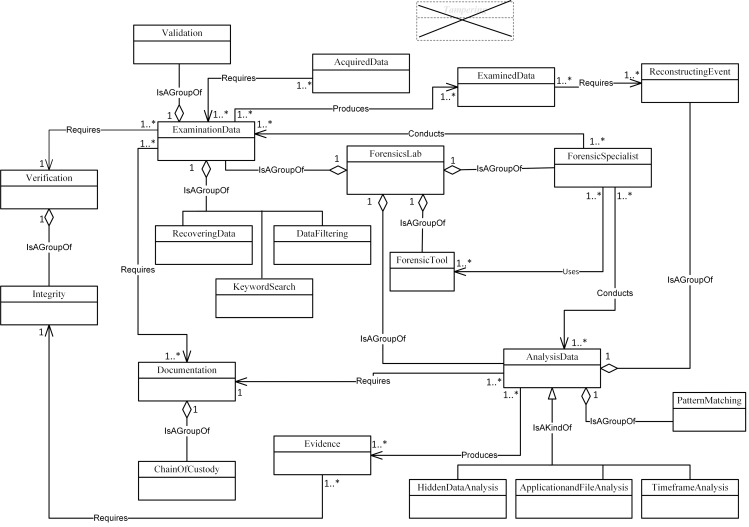
A validated version of examination & analysis -phase class of concepts.

**Fig 15:**
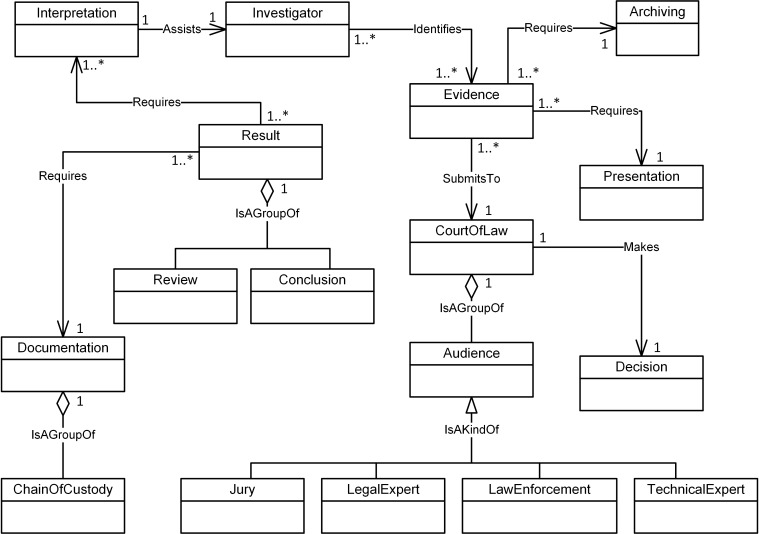
A validated version of reporting -phase class of concepts.

Because of frequency-based selection, classes for the Preservation and Examination & Analysis phases have been changed, whereas the classes for Acquisition and Reporting phases remain unchanged. Figs 12–15 show the last version of our MFM named MFM1.2.

Many people who are directly (e.g.: forensic investigators, cybersecurity agencies, police officers) or indirectly (e.g.: law enforcement agencies, IT companies) involved in mobile forensic operations generally do not have a complete view of how different mobile forensic activities can be conducted. MFM through its four sets of classes (preservation, acquisition, examination & analysis and reporting) can provide a picture of how all mobile forensic actions should be performed. Additionally, the developed metamodel contributes to the facilitation of sharing MF knowledge. It presents a new a metamodeling-based approach to guide mobile forensics practitioners on how to conduct mobile forensics investigation process properly. This is a specific artifact to describe a mobile forensics language. As the MFM has the ability to offer a modelling guideline to many domain users, various users can quickly find decision solutions from semantic models. Moreover, the resultant metamodel provides investigators with logical and sensible investigation concepts that may be needed during investigation process. Most of the concepts and terminologies of the mobile forensics domain were used in the MFM.

## 4. Conclusion

The issues and challenges of mobile forensics investigation have been presented and discussed through this paper. Based on our observation, the lack of knowledge management in mobile forensics has led to a certain problems in this domain. These are i) the difficulty of investigation for new investigators, ii) ambiguity in mobile forensics’ concepts and terminologies and iii) the difficulty in understanding the various processes involved in this domain. To overcome these issues, the metamodeling approach has been selected and discussed briefly in this paper. We used 21 models (Set1) for the initial development of MFM. In the second iteration, 10 models (Set V1) were used for validation (using the technique of comparison against other models) to identify any missing concepts in the initial version of the metamodel and to ensure its broad coverage. In the third iteration, we used another 10 models (Set V2) for a second validation (using frequency-based selection) to evaluate the importance of individual concepts. These two validations improved the expressiveness and the completeness of the concepts in MFM. Our MFM contributes to the increase of knowledge for both internal and external stakeholders in the digital forensics domain. Through the MFM, the artifact is hoped to help increase the efficiency of mobile forensic investigation in various forensic agencies. The MFM presents all the required concepts that could assist the designers in modelling all respective aspects when designing a mobile forensic enabled system and service.

Our future work based on results gathered from this paper is to continue to develop a repository based on the MFM to store MF knowledge and to allow a responsive and flexible MF approach.

## Supporting information

S1 Table Selection of common concepts. (DOCX)

S2 Table Definitions of MFM concepts. (DOCX)

S3 Table Validation summary against model set V1. (DOCX)

S4 Table Validation summary against model Set V2. (DOCX)
