# Supplementary material for: A metamodel for mobile forensics investigation domain
Source: PLoS One. 2017 Apr 26;12(4):e0176223. doi: 10.1371/journal.pone.0176223 (PMC5433730; doi:10.1371/journal.pone.0176223)
Supplement: S1 Table — (DOCX) [file pone.0176223.s001.docx]

**S1 Table. Selection of Common Concepts**

| **No** | **Common Concept** | **Concepts** | **Frequency** | **Generality** | **Definition** |
| --- | --- | --- | --- | --- | --- |
| 1 | Investigation Procedure | Investigation Procedure | 9 | 1 | 1 |
| 2 | Chain of Custody | Chain of Custody | 9 | 1 | 1 |
| 3 | Crime | Incident | 2 | 1 | 1 |
|  |  | Case | 1 | 0 | 0 |
|  |  | Crime | 6 | 1 | 1 |
|  | Identification | Identification | 8 | 1 | 1 |
|  |  | Recognition | 2 | 1 | 1 |
|  |  | Classification | 2 | 1 | 1 |
| 5 | Legal Authority | Legal Authority | 3 | 1 | 1 |
|  |  | Authority | 2 | 1 | 1 |
|  |  | Legal Jurisdiction | 1 | 0 | 1 |
|  |  | Jurisdictional Law | 1 | 1 | 0 |
|  |  | Jurisdiction | 1 | 1 | 1 |
|  |  | legislation | 1 | 1 | 1 |
| 6 | Search Warrant | Search Warrant | 6 | 1 | 1 |
|  |  | Warrant | 1 | 0 | 1 |
| 7 | External Storage | Removable Media | 2 | 1 | 1 |
|  |  | Removable Component | 1 | 0 | 0 |
|  |  | Removable Data Storage | 1 | 0 | 0 |
|  |  | External Storage Media | 1 | 1 | 1 |
|  |  | External Storage | 4 | 1 | 1 |
|  |  | External Memory | 2 | 1 | 0 |
| 8 | Mobile Device | Mobile Device | 8 | 1 | 1 |
|  |  | Suspect Device | 1 | 0 | 0 |
|  |  | Hand-held device | 1 | 1 | 1 |
|  |  | Device | 2 | 1 | 0 |
|  |  | Target Device | 1 | 0 | 0 |
| 10 | Potential Evidence | Potential Evidence | 8 | 1 | 1 |
| 11 | Forensic Tool | Forensic Technique | 1 | 1 | 0 |
|  |  | Technique | 1 | 0 | 0 |
|  |  | Forensic Tool | 7 | 1 | 1 |
|  |  | Tool | 6 | 1 | 0 |
|  |  | Toolkit | 1 | 0 | 0 |
| 12 | Documentation | Documentation | 11 | 1 | 1 |
| 13 | Preparation | Preparation | 11 | 1 | 1 |
| 14 | Isolation | Isolation | 4 | 1 | 1 |
|  |  | Radio Frequency Isolation | 1 | 0 | 0 |
|  |  | Signal Isolation | 1 | 0 | 0 |
|  |  | Communication Shielding | 3 | 1 | 1 |
|  |  | Cut Network Communication | 1 | 0 | 0 |
|  |  | Disable Signal | 1 | 0 | 0 |
| 15 | Faraday Bag | Faraday Bag | 4 | 1 | 1 |
|  |  | Faraday Cage | 2 | 0 | 0 |
|  |  | Radio Frequency Shielding | 1 | 1 | 0 |
| 16 | Extraction | Extraction | 7 | 1 | 1 |
| 17 | Physical Acquisition | Physical Memory Dump | 1 | 0 | 0 |
|  |  | Physical Acquisition | 7 | 1 | 1 |
|  |  | Physical Collection | 1 | 0 | 0 |
|  |  | Physical Forensic Image | 1 | 0 | 0 |
| 18 | Logical Acquisition | Logical Collection | 1 | 0 | 0 |
|  |  | Logical Acquisition | 8 | 1 | 1 |
| 19 | Manual Acquisition | Manual Extraction | 2 | 0 | 1 |
|  |  | Manual Acquisition | 4 | 1 | 1 |
| 20 | Forensic Examiner | Forensic Examiner | 5 | 1 | 1 |
|  |  | Analyst | 1 | 1 | 1 |
|  |  | Forensic Analyst | 2 | 1 | 1 |
| 21 | Verification | Verification | 4 | 1 | 1 |
| 22 | Hashing | Hash Value | 2 | 0 | 1 |
|  |  | Hashing Technique | 2 | 0 | 1 |
|  |  | Hashing Method | 1 | 0 | 0 |
|  |  | Hashing Algorithm | 1 | 0 | 0 |
|  |  | Hashing | 6 | 1 | 1 |
|  |  | Hash Function | 1 | 0 | 0 |
| 23 | Integrity | Integrity | 13 | 1 | 1 |
|  |  | Data Integrity | 1 | 0 | 0 |
| 24 | Presentation | Presentation | 17 | 1 | 1 |
| 25 | Court of Law | Court | 4 | 1 | 1 |
|  |  | Court of Law | 5 | 1 | 1 |
|  |  | Court Ruling | 1 | 0 | 0 |
| 26 | Investigator | Investigator | 14 | 1 | 1 |
|  |  | Forensic Practitioner | 1 | 1 | 0 |
|  |  | Practitioner | 1 | 0 | 0 |
|  |  | Forensic Investigator | 2 | 1 | 0 |
| 27 | Audience | Audience | 5 | 1 | 1 |
|  |  | Stakeholder | 1 | 1 | 0 |
| 28 | Evidence | Evidence | 19 | 1 | 1 |
| 29 | Pattern Matching | Pattern Matching | 4 | 1 | 1 |
| 30 | Interpretation | Interpretation | 5 | 1 | 1 |
|  |  | Explaining | 1 | 0 | 0 |
| 31 | Review | Review | 5 | 1 | 1 |
| 32 | Result | Result | 9 | 1 | 1 |
|  |  | Finding | 3 | 1 | 0 |
| 33 | Crime Scene | Crime Scene | 9 | 1 | 1 |
|  |  | Scene | 3 | 1 | 1 |
| 34 | Authorization | Authorization | 6 | 1 | 1 |
| 35 | People | People | 5 | 1 | 1 |
| 36 | Packaging and Sealing | Packaging and Sealing | 4 | 1 | 1 |
| 37 | Transportation and Storage | Transportation and Storage | 9 | 1 | 1 |
| 38 | Planning | Search Plan | 1 | 1 | 0 |
|  |  | Planning | 3 | 1 | 1 |
|  |  | Investigation Plan | 1 | 1 | 0 |
| 39 | Shock | Shock | 3 | 1 | 1 |
| 40 | Humidity | Humidity | 3 | 1 | 1 |
| 41 | Temperature | Temperature | 3 | 1 | 1 |
| 42 | Victim | Victim | 3 | 1 | 1 |
| 43 | Suspect | Suspect | 6 | 1 | 1 |
|  |  | Accused | 1 | 1 | 0 |
| 44 | Witness | Witness | 3 | 1 | 1 |
| 45 | Forensic Specialist | Forensic Specialist | 4 | 1 | 1 |
| 46 | Volatile Evidence | Volatile Evidence | 6 | 1 | 1 |
|  |  | Volatile Data | 1 | 0 | 1 |
| 47 | Non-Volatile Evidence | Non-Volatile Evidence | 4 | 1 | 1 |
| 48 | Acquired Data | Acquired Data | 5 | 1 | 1 |
|  |  | Collected Evidence | 3 | 1 | 1 |
|  |  | Collected Data | 3 | 1 | 0 |
| 49 | Data Filtering | Data Filtering | 3 | 1 | 1 |
|  |  | Filtering | 3 | 1 | 1 |
| 50 | Validation | Validation | 5 | 1 | 1 |
| 51 | Tampering | Tampering | 3 | 1 | 1 |
| 52 | Recovering Data | Recovering Data | 5 | 1 | 1 |
|  |  | Retrieved Data | 3 | 1 | 0 |
|  |  | Retrieved Evidence | 1 | 1 | 0 |
| 53 | Reconstructing Event | Reconstructing Event | 5 | 1 | 1 |
|  |  | Reconstructing Scene | 1 | 1 | 0 |
| 54 | Timeframe Analysis | Timeframe Analysis | 4 | 1 | 1 |
| 55 | Hidden Data Analysis | Hidden Data Analysis | 5 | 1 | 1 |
| 56 | Application and File Analysis | Application and File Analysis | 3 | 1 | 1 |
| 57 | Law Enforcement | Law Enforcement | 3 | 1 | 1 |
|  |  | Law Enforcement Official | 1 | 1 | 0 |
|  |  | Law Enforcement Agency | 2 | 1 | 0 |
| 58 | Technical Expert | Technical Expert | 2 | 1 | 1 |
|  |  | Technical Review | 1 | 1 | 1 |
| 59 | Legal Expert | Legal Expert | 4 | 1 | 1 |
| 60 | Conclusion | Conclusion | 6 | 1 | 1 |
| 61 | Jury | Jury | 4 | 1 | 1 |
|  |  | judge | 1 | 1 | 0 |
| 62 | Recording | Recording | 5 | 1 | 1 |
|  |  | Recorded Information | 1 | 0 | 0 |
|  |  | Recording Scene | 1 | 1 | 1 |
| 63 | Photographing | Photographing | 9 | 1 | 1 |
| 64 | Sketching | Sketching | 3 | 1 | 1 |
|  |  | Crime scene Mapping | 1 | 1 | 1 |
| 65 | Investigation Strategy | Systematic Strategy | 2 | 1 | 0 |
|  |  | Investigation Strategy | 2 | 1 | 1 |
|  |  | Response Strategy | 1 | 1 | 1 |
| 66 | Forensics Lab | Laboratory | 3 | 1 | 1 |
|  |  | Laboratory Evidence | 1 | 1 | 1 |
|  |  | Forensics Lab | 3 | 1 | 1 |
|  |  | Forensic Laboratory | 2 | 1 | 1 |
| 67 | Securing Scene | Securing Scene | 6 | 1 | 1 |
| 68 | Acquisition Method | Acquisition Method | 3 | 1 | 1 |
| 69 | Backup | Backup | 6 | 1 | 1 |
|  |  | Copy of Evidence | 3 | 1 | 0 |
| 70 | First Responder | First Responder | 4 | 1 | 1 |
| 71 | Equipment | Equipment | 6 | 1 | 1 |
| 72 | Decision | Decision | 4 | 1 | 1 |
|  | EnvironmentalEffect | EnvironmentalEffect | 3 | 1 | 1 |
| 73 |  | Circumstances | 1 | 0 | 0 |
|  |  | Environmental Circumstances | 1 | 1 | 0 |
| 74 | Internal Memory | Internal Memory | 6 | 1 | 1 |
| 75 | KeywordSearch | KeywordSearch | 5 | 1 | 1 |
| 76 | Source | Source | 9 | 1 | 1 |
| 77 | Bootloader | Bootloader | 4 | 1 | 1 |
| 78 | Airplane mode | Airplane mode | 4 | 1 | 1 |
| 79 | Cell Site Analysis | Cell Site Analysis | 2 | 1 | 1 |
| 80 | Network Provider | Network Provider | 4 | 1 | 1 |
|  |  | local service provider | 1 | 1 | 0 |
|  |  | cellular provider | 1 | 0 | 0 |
| 81 | Rooting | Rooting | 4 | 1 | 1 |
| 82 | Archiving | Archiving | 1 | 1 | 1 |
