# Supplementary material for: A metamodel for mobile forensics investigation domain
Source: PLoS One. 2017 Apr 26;12(4):e0176223. doi: 10.1371/journal.pone.0176223 (PMC5433730; doi:10.1371/journal.pone.0176223)
Supplement: S2 Table — (DOCX) [file pone.0176223.s002.docx]

**S2 Table. Definitions of MFM Concepts**

| **MFM Concepts** | | **Definition** |
| --- | --- | --- |
| ***Preservation Concept*** | | |
| 1 | InvestigationProcedure | Legal steps which follow by investigators during investigation processes (preservation, acquisition, examination, analysis, and reporting) |
| 2 | ChainofCustody | Is a process that tracks the movement of evidence through its collection, preservation, and analysis lifecycle by documenting each person who handled the evidence, the date/time it was collected or transferred |
| 3 | Crime | Illegal activities such as money laundering, harassment, trafficking of child pornography, trafficking drugs, fraud and identity theft using mobile devices |
| 4 | Identification | A process that used by investigator to identify type of mobile device and its operating system, people in the crime scene, external data storage and potential evidence sources |
| 5 | LegalAuthority | The investigators must have consent from legal authority for conducting investigation |
| 6 | MobileDevice | Has a high storage capacity that provides communications, digital photography, navigation systems, entertainment, data storage, and personal information |
| 7 | SearchWarrant | A warrant issued by the legal authority authorizing investigators to search a specified place for evidence |
| 9 | PotentialEvidence | Includes a Voice Communication(Outgoing, incoming, missed call), Text messages( e-mail, instant messaging and short text messaging (SMS) ), Wi-Fi network, Web browsing activities, Electronic documents, Social media related data ,Application related data, Location information ( GPS ) |
| 10 | Documentation | Is a continuous activity that required in all the stages and used for documenting the crime Scene (Photographing, Sketching, and Recording) |
| 11 | Preparation | Is a process that occurs prior to the actual investigation that involves an initial understanding of the nature of the crime and activities like preparing the tools required for standard portable electronic device investigations, building an appropriate team, assigning roles to each personnel and accumulating materials for packing evidence sources |
| 12 | Isolation | Is a process that used to isolate mobile device from communication sources (Wi-Fi network, Bluetooth) to prevent the addition of new data to the phone through incoming calls and text messages as well as the potential destruction of data through remote access or remote wiping |
| 13 | FaradayBag | is an enclosure used in order to prevent signals such as Wi-Fi, Bluetooth and GSM from connecting with the mobile device during transportation to the forensic laboratory |
| 14 | CrimeScene | The location (spot) of a crime |
| 15 | Authorization | The right of access to evidence, that is obtained from authorities for investigators to extract potential digital evidence from the mobile device |
| 16 | People | Who are related to the crime which includes victims, suspects, bystanders, witnesses and forensic personnel |
| 17 | PackagingAndSealing | A process that used for sealing the evidence to protect it from electromagnetic radiations, dust, heat and moisture |
| 18 | Transportation | A process of transferring digital evidence from the crime scene to the laboratory forensic. |
| 19 | Storage | The evidence should be stored in a secure place, protected from electromagnetic radiations, dust, heat, moisture and unauthorized people should not have access to the storage place |
| 20 | Planning | An initial understanding of the crime scene and activities such preparing the tools required for mobile device, building an appropriate team, assigning roles to each forensic personnel |
| 21 | Shock | Is an environmental effect that may destroy the evidence during their transportation to the forensic lab from the crime scene |
| 22 | Humidity | Has an adverse effect on the devices, hence the special arrangements may be required to avoid environmental effects |
| 23 | Temperature | Is an environmental effect that may destroy the digital evidence during transportation to the forensic lab |
| 24 | Victim | A person who has had something bad happen to him |
| 25 | Suspect | An individual who is suspected of committing a crime |
| 26 | Witness | A person who sees an event and reports what happened; a person called upon by either side in a lawsuit to give testimony before the court or jury |
| 27 | Collection | A process for collecting evidence from crime scene by forensic investigators |
| 28 | Recording | A process for helping in recreating the crime scene and reviewing it any time, preserving the chain of potential evidence and the preparation for the investigation findings presentation |
| 29 | Photographing | A process for making photographs for crime scene and all the electronic devices (mobile device, adaptors, cables etc.), may be these photographs useful, especially for court presentation purposes |
| 30 | Sketching | A process for drawing location of crime scene and the relationship of evidentiary items to the surroundings |
| 31 | InvestigationStrategy | Is an approach for carrying out the investigation to maximize the collection of potential evidence |
| 32 | AirplaneMode | which is designed to disable the device’s radios |
| 33 | Rooting | is a mandatory requirement in Android forensics as it allows access to the root folder of the Android file system where all the evidence is, installing external binaries |
| 34 | ForensicsLab | Is a special place that used to extract evidence from mobile devices and analyze it |
| 35 | UnlockBootloader | Is a little bit of code that tells your device's operating system how to boot up |
| 36 | SecuringScene | Is A process that used for preventing the contamination and corruption of evidence and secure the crime scene from unauthorized access |
| 37 | CellSiteAnalysis | Is a legally accepted process which identifies the location and movement of a mobile phone over a period time |
| 38 | FirstResponder | Identifies the evidence and list of components (phone, its charger, the memory card and the USB cable) that may be found at the crime scene |
| 39 | Equipment | Includes media, cables, faraday bags and power adapters |
| 40 | Network provider | Is a civilian provider who has completed the credentialing process and signed a contracted agreement to be part of the network of providers |
| 41 | Hypothesis | Gives an idea to the investigator what evidence that must be collected and he can choose the appropriate tool which will be according to mobile phone |
| 42 | EnvironmentalEffect | Are natural effects which cause damage to the digital evidence during transportation to the forensic lab |
| ***Acquisition Concept*** | | |
| 1 | ExternalStorage | A set of devices such as MMC cards, Compact Flash (CF) cards, memory sticks, Secure Digital (SD) cards, USB memory sticks etc. These devices contain non-volatile evidence |
| 2 | ForensicTool | Typically designed to collect, acquire, analyze and examine digital evidence |
| 3 | Documentation | Is a continuous activity that required in all the stages and used for documenting the acquisition process. |
| 4 | ChainofCustody | Is a process that tracks the movement of evidence through its collection, preservation, and analysis lifecycle by documenting each person who handled the evidence, the date/time it was collected or transferred |
| 5 | Extraction | Is a process to acquire data from mobile phone using acquisition methods which are manual acquisition, logical acquisition, and physical acquisition |
| 6 | PhysicalAcquisition | Is a method that used to assist the examiner to search the contents of the removable media and potentially recover deleted files |
| 7 | LogicalAcquisition | Is a method that used to obtain user data (SMS, call logs, pictures, video, contacts, calendar etc.) that reside on logical store (e.g. file system partition) |
| 8 | ManualAcquisition | Is a method that based on observing the mobile device user interface and fully extraction of evidence when other techniques are not possible |
| 9 | ForensicExaminer | Has ability to gather information about the individuals, determine the exact nature of the events that occurred, construct a timeline of events, uncover information that explains the motivation for the offense and discover what tools are used |
| 10 | VolatileEvidence | Is a data which be prone to destruction when mobile device state is changed or with the loss of power |
| 11 | Non-VolatileEvidence | Is a data which be in the non- volatile memory of mobile such as flash memory (NAND) and external memory (SD), these data are not affected by a loss of power |
| 12 | AcquiredData | Is a data that produced from manual, logical and physical acquisition |
| 13 | ForensicsLab | Is a special place that used to extract evidence from mobile devices and analyze it |
| 14 | Backup | Before examination of the evidence, extra copies of the acquired evidence should be made. |
| 15 | InternalMemory | Is a flash memory (NAND) that includes components of the mobile device such as SMS, call logs, photos, MMS, email, videos, calendar note, audio, documents, MMS etc |
| 16 | Imaging | Is a process that uses software to copy of all electronic data on a device, performed in a manner that ensures the information is not altered |
| 17 | MobileDevice | Has a high storage capacity that provides communications, digital photography, navigation systems, entertainment, data storage, and personal information |
| 18 | AcquisitionMethod | Is divided into three categories: manual, logical and physical which use for obtaining data from a mobile device |
| 19 | ExternalStorage | It includes MMC cards, Compact Flash (CF) cards, memory sticks, Secure Digital (SD) cards, USB memory sticks etc. These devices contain non-volatile evidence |
| 20 | Hashing | Is a process that used to maintain the integrity of acquired evidence through using a mathematical algorithm (e.g. MD5, Secure Hash Algorithm (SHA)) against data to produce a numeric value that is representative of that data |
| 21 | Integrity | Is a process that used for ensuring that the evidence collected has not tampered with it till tested and presented to the court |
| ***Examination &Analysis Concept*** | | |
| 1 | Verification | Is a process that used to verify the integrity of the data extracted from the phone through verification techniques like hashing method |
| 2 | Integrity | Is a process that used for ensuring that the evidence collected has not tampered with it till tested and presented to the court |
| 3 | PatternMatching | Using to help digital forensics examiner to search for potential evidence on mobile devices systematically |
| 4 | ForensicSpecialist | Is a member of investigation team who locates, identifies, collects, analyses and examines data while preserving the integrity and maintaining a strict chain of custody of information discovered |
| 5 | DataFiltering | Is a process that requires for huge volumes of acquired data to be converted into a manageable size for future analysis purpose |
| 6 | AcquiredData | Is a data that produced from manual, logical and physical acquisition |
| 7 | Validation | Is a process that conducted by forensic examiner to check if data extracted from the mobile device matches to original data of device itself |
| 8 | RecoveringData | Recovering erased files and data from mobile device for conducting forensic investigation properly |
| 9 | ReconstructingEvent | Is a process of determining the events that occurred at a crime scene using evidence characteristics |
| 10 | TimeFrameAnalysis | Determines when events occurred on the system to associate usage with an individual by reviewing any logs present and the date/time stamps in the file system, like the last modified time |
| 11 | HiddenDataAnalysis | Detect and recover hidden data that may indicate knowledge, ownership, or intent by correlating file headers to file extensions to show intentional obfuscation; gaining access to password-protected, encrypted, and compressed files; gaining access to stenographic information detected in images; and gaining access to reserved areas of data storage outside the normal file system |
| 12 | ApplicationandFile Analysis | Identify information relevant to the investigation by examining file content, correlating files to installed applications, identifying relationships between files (email files to email attachments), determining the significance of unknown file types, examining system configuration settings, and examining file metadata (documents containing authorship identification) |
| 13 | ForensicsLab | Is a special place that used to extract evidence from mobile devices and analyze it |
| 14 | Evidence | Is an electronic information stored or transmitted in binary form which resides on internal memory( address book, E-mail, Browser history, SMS, Media etc. ), SIM card ( PIN code, PUK code, IMEI, IMSI) and external memory cards, this evidence may be relied on in the court |
| 15 | AnalysisData | Is a process for identifying relationships between fragments of data, analyzing hidden data, identifying the importance of the information acquired from the examination phase |
| 16 | ExaminationData | Is a process that uncovers digital evidence which may be hidden or obscure |
| 17 | ForensicTool | Typically designed to collect, acquire, analyze and examine digital evidence |
| 18 | Documentation | Is a continuous activity that required in all the stages and used for documenting the Examination &Analysis process |
| 19 | ChainOfCustody | Is a process that tracks the movement of evidence through its collection, preservation, and analysis lifecycle by documenting each person who handled the evidence, the date/time it was collected or transferred |
| 20 | DataExamined | The output of examination process |
| 21 | KeywordSearch | Is a method used to identify evidence |
| ***Reporting Concept*** | | |
| 1 | Presentation | The investigator presents the investigation results to the court through description the sequence of events conducted in the analysis phase, shows how the evidence is linked to locations and particular dates/times |
| 2 | CourtOfLaw | Is a governmental institution that hears cases and makes decisions based on statutes or the common law |
| 3 | Investigator | Is a member of investigation team who has a responsibility to gather data about the suspect or person of interest such as physical addresses, known aliases, prior criminal history, and education level, relationship to the victim, list of electronic devices owned and he responsible for the control of the crime scene by defining the boundaries of the crime and controlling the gathered crowd over there |
| 4 | Audience | The audience includes law enforcement officials, technical experts, legal experts, jury, and corporate management, the final result of investigations presents to them |
| 5 | Evidence | Is an electronic information stored or transmitted in binary form which resides on internal memory( address book, E-mail, Browser history, SMS, Media etc. ), SIM card ( PIN code, PUK code, IMEI, IMSI) and external memory cards, this evidence may be relied on in the court |
| 6 | Interpretation | Is a process that utilizes scientifically proven methods and techniques to explain facts found during the digital evidence examination and analysis process |
| 7 | Review | Is a process for reviewing all the steps in the investigation process and identifying areas which require improvement |
| 8 | Result | Is the final findings of forensic investigation which include all the information necessary to identify the crime and its source |
| 9 | LawEnforcement | Is a government agency responsible for the enforcement of the laws |
| 10 | TechnicalExpert | Provides specific knowledge |
| 11 | LegalExpert | Legal consultant |
| 12 | Conclusion | Is a summarization of investigation results |
| 13 | Jury | Gives a judgment in a legal case based on evidence submitted to court |
| 14 | Decision | Decision of the court as to what to do with the evidence is made and close the case |
| 15 | ChainOfCustody | Is a process that tracks the movement of evidence through its collection, preservation, and analysis lifecycle by documenting each person who handled the evidence |
| 16 | Documentation | Is a continuous activity that required in all the stages and used for documenting the reporting process |
| 17 | Archiving | Is a necessary process to retain the data in a useable format for the ongoing court process, future reference, and for record keeping requirements |
