# Supplementary material for: A metamodel for mobile forensics investigation domain
Source: PLoS One. 2017 Apr 26;12(4):e0176223. doi: 10.1371/journal.pone.0176223 (PMC5433730; doi:10.1371/journal.pone.0176223)
Supplement: S3 Table — (DOCX) [file pone.0176223.s003.docx]

**S3 Table Validation summary against Model Set V1**

| **Model Set V1 Concept** | **MFM1.0 concept** |
| --- | --- |
| ***Model 1*** |  |
| Extraction | Extraction |
| Android Device | MobileDevice |
| Acquisition Process | AcquisitionMethod |
| Integrity | Integrity |
| Copy File | Backup |
| Faraday Cage | Faraday Bag |
| Shielding Device | Isolation |
| Acquired Data | AcquiredData |
| Hashing | Hashing |
| Verification | Verification |
| LawEnforcement | Law Enforcement |
| Documentation | Documentation |
| Evidence | Evidence |
| ***Model 2*** |  |
| Incident | Crime |
| Archiving | *Not supported (add to Reporting class)* |
| Investigator | Investigator |
| Evidence | Evidence |
| EvidenceExtraction | Extraction |
| Analysis | AnalysisData |
| Authority | LegalAuthority |
| Examination | ExaminationData |
| Recovering | Recovering |
| Examiner | ForensicSpecialist |
| DataIntegrity | Integrity |
| Physical Acquisition | PhysicalAcquisition |
| Logical Acquisition | LogicalAcquisition |
| Rooting | Rooting |
| TowerData | CellSiteAnalysis |
| Internal Memory | InternalMemory |
| Copy | Backup |
| Location | CrimeScene |
| Event | Crime |
| TimestampInformation | TimeframeAnalysis |
| Hash Value | Hashing |
| ApplicationAnalysis | ApplicationAnalysis |
| Result | Result |
| Verdict | Decision |
| Technique | ForensicTool |
| ***Model 3*** |  |
| Evidence | Evidence |
| ForensicPractitioner | ForensicSpecialist |
| Procedure | Procedure |
| Interpretation | Interpretation |
| Finding | Result |
| KeywordSearch | KeywordSearch |
| Hashing | Hashing |
| Integrity | Integrity |
| Analysis | AnalysisData |
| Rooting | Rooting |
| Target Device | MobileDevice |
| Forensic Examination | ExaminationData |
| Bootloader | Bootloader |
| Data Examined | ***Not supported (add to Examination & Analysis class)*** |
| Identification | Identification |
| External Storage | ExternalStorage |
| Presentation | Presentation |
| Court of Law | CourtofLaw |
| Internal Storage | InternalMemory |
| Imaging | ***Not supported (add to Acquisition)*** |
| ***Model 4*** |  |
| Forensics Tool | ForensicsTool |
| Analysis | AnalysisData |
| Conclusion | Conclusion |
| Acquired Data | AcquiredData |
| Mobile Phone | MobileDevice |
| ***Model 5*** |  |
| Forensic Investigator | Investigator |
| Mobile Phone | MobileDevice |
| Evidence | Evidence |
| Integrity | Integrity |
| Imaging | ***Not supported (add to Acquisition)*** |
| Forensic Tool | ForensicTool |
| Evidence Extracted | AcquiredData |
| Extraction | Extraction |
| Evidence Analysis | AnalysisData |
| Shielding | Isolation |
| Transporting and Storing | TransportingAndStoring |
| Recording | Recording |
| Archiving | *Not supported (add to Reporting class)* |
| Hash Algorithm | Hashing |
| Identification | Identification |
| Procedure | Procedure |
| Documentation | Documentation |
| Recovering | Recovering |
| Report Conclusion | Conclusion |
| ***Model 6*** |  |
| Evidence | Evidence |
| Analysis | AnalysisData |
| Tool | ForensicTool |
| Procedure | Procedure |
| Integrity | Integrity |
| Investigator | Investigator |
| ServiceProvider | NetworkProvider |
| Extracting Data | Extracting |
| Logical Collection | LogicalAcquisition |
| Rooting | Rooting |
| Bootloader | Bootloader |
| Mobile Device | MobileDevice |
| ***Model 7*** |  |
| Preparation | Preparation |
| Investigator | Investigator |
| Forensic Tool | ForensicTool |
| Evidence | Evidence |
| Acquired Data | AcquiredData |
| Documentation | Documentation |
| Analysis | AnalysisData |
| Internal Storage | InternalMemory |
| Mobile Device | MobileDevice |
| ***Model 8*** |  |
| Data Extraction | Extracting |
| Logical Acquisition | LogicalAcquisition |
| Imaging | ***Not supported (add to Acquisition)*** |
| Analysis | AnalysisData |
| Mobile Device | MobileDevice |
| Physical Acquisition | PhysicalAcquisition |
| Manual Acquisition | ManualAcquisition |
| Forensic Tool | ForensicTool |
| Integrity | Integrity |
| Acquired Data | AcquiredData |
| Investigator | Investigator |
| Validation | Validation |
| ***Model 9*** |  |
| Investigator | Investigator |
| Suspect | Suspect |
| Evidence Transmission | TransportingAndStorage |
| Integrity | Integrity |
| Location | CrimeScene |
| Evidence Storage | TransportingAndStorage |
| Validation | Validation |
| Hypothesis | ***Not supported (add to class Preservation)*** |
| Court of Law | CourtOfLaw |
| Authorization | Authorization |
| Authority | LegalAuthority |
| CellPhoneProvider | NetworkProvider |
| Presentation | Presentation |
| Suspected Crime | Crime |
| Potential Evidence | Potential Evidence |
| ***Model 10*** |  |
| Hypothesis | ***Not supported (add to class Preservation)*** |
| Crime Scene | CrimeScene |
| Device Determination | Identification |
| Data Extraction | Extraction |
| Logical Extraction | Logical Acquisition |
| Physical Extraction | Physical Acquisition |
| Documentation | Documentation |
| Tool | ForensicTool |
| Evidence | Evidence |
| Procedure | Procedure |
| Investigator | Investigator |
| Volatile Data | Volatile Evidence |
| Internal Memory | Internal Memory |
| External Memory | External Storage |
| Crime | Crime |
| Judicial Authority | Legal Authority |
| Suspect | Suspect |
