# Supplementary material for: A metamodel for mobile forensics investigation domain
Source: PLoS One. 2017 Apr 26;12(4):e0176223. doi: 10.1371/journal.pone.0176223 (PMC5433730; doi:10.1371/journal.pone.0176223)
Supplement: S4 Table — (DOCX) [file pone.0176223.s004.docx]

**S4 Table**

**Validation summary against Model Set V2**

| **Model Set V2 Concept** | **MFM1.1 Concept** |
| --- | --- |
| ***Model 1*** |  |
| Documentation | Documentation |
| Authorization | Authorization |
| Chain of Custody | ChainofCustody |
| Evidence | Evidence |
| Incident | Crime |
| First Response | FirstResponse |
| Investigator | Investigator |
| Planning | Planning |
| Forensic Tool | ForensicTool |
| Preparation | Preparation |
| Equipment | Equipment |
| Incident Scene Location | CrimeScene |
| Recording | Recording |
| Photographing | Photographing |
| Potential Evidence | PotentialEvidence |
| Identiﬁcation | Identiﬁcation |
| Integrity | Integrity |
| Presentation | Presentation |
| Evidence Examined | ExaminedData |
| Examination Evidence | ExaminationData |
| Collection | ***Not supported* (add to class Preservation)** |
| Faraday Bag | FaradayBag |
| Procedure | Procedure |
| Transportation | TransportationAndStorage |
| Evidence Storage | TransportationAndStorage |
| Evidence Analysis | AnalysisData |
| Hypothesis | Hypothesis |
| Reconstructing the Scene | ReconstructingEvent |
| Decision | Decision |
| Evidence Interpretation | Interpretation |
| Court | CourtofLaw |
| Backup | Backup |
| Stakeholder | Audience |
| ***Model 2*** |  |
| Procedure | Procedure |
| Preparation | Preparation |
| Internal Memory | InternalMemory |
| Forensic Tool | ForensicTool |
| Integrity | Integrity |
| Packaging | Packaging |
| Verification Data | Verification |
| Imaging | Imaging |
| Data Acquired | DataAcquired |
| Documentation | Documentation |
| Physical Acquisition | PhysicalAcquisition |
| Logical Acquisition | LogicalAcquisition |
| Finding | Result |
| Analysis | AnalysisData |
| Examination | ExaminationData |
| Mobile Device | MobileDevice |
| Forensic Investigator | Investigator |
| ***Model 3*** |  |
| Forensic Analyst | ForensicSpecialist |
| Acquisition Method | AcquisitionMethod |
| Procedure | Procedure |
| Mobile Device | MobileDevice |
| Evidence | Evidence |
| Documentation | Documentation |
| Equipment | Equipment |
| Extracting Data | Extracting |
| Internal Memory | InternalMemory |
| Copying Data | Backup |
| Forensic Tool | ForensicTool |
| Hash | Hashing |
| Isolation | Isolation |
| Examination | ExaminationData |
| Examiner | Forensic Examiner |
| Acquired Data | AcquiredData |
| Imaging | Imaging |
| Recovering | RecoveringData |
| KeywordSearch | KeywordSearch |
| Law Enforcement | LawEnforcement |
| Physical Extraction | PhysicalAcquisition |
| Forensic Expert | ForensicSpecialist |
| Suspect | Suspect |
| Manual Extraction | ManualAcquisition |
| Analysis Application | AnalysisFileAndApplication |
| Analysis Data | AnalysisData |
| ***Model 4*** |  |
| Preparation | Preparation |
| Crime | Crime |
| Authorization | Authorization |
| Search Warrant | SearchWarrant |
| Securing the Scene | SecuringScene |
| Identification | Identification |
| Documentation | Documentation |
| Reconstruction | ReconstructionEvent |
| Hypothesis | Hypothesis |
| Packaging | Packaging |
| Forensic Laboratory | ForensicLab |
| Extracting | Extraction |
| Mobile Device | MobileDevice |
| Forensic Tool | ForensicTool |
| Suspect | Suspect |
| Victim | Victim |
| Data recovery | RecoveringData |
| Interpretation | Interpretation |
| Result | Result |
| Jury | Jury |
| Presentation | Presentation |
| Review | Review |
| People | People |
| Investigator | Investigator |
| Court of Law | CourtOfLaw |
| Evidence | Evidence |
| Ruling | Decision |
| Volatile Evidence | VolatileEvidence |
| Evidence Analysis | AnalysisData |
| Evidence Examination | ExaminationData |
| Evidence Acquired | EvidenceAcquired |
| Crime Scene | CrimeScene |
| Sketching | Sketching |
| Photographing | Photographing |
| Authority | LegalAuthority |
| Witness | Witness |
| Device Isolation | Isolation |
| ***Model 5*** |  |
| Evidence | Evidence |
| Forensics Investigator | Investigator |
| Smartphone Device | MobileDevice |
| Crime Scene | CrimeScene |
| Documentation | Documentation |
| Packaging | Packaging |
| Faraday Container | FaradayBag |
| Smartphone Device Isolation | Isolation |
| Suspect | Suspect |
| Transportation | Transportation |
| Forensic Lab | ForensicLab |
| Collection | ***Not supported*** *(add to class* ***Preservation)*** |
| Potential Evidence | PotentialEvidence |
| Forensic Specialist | ForensicSpecialist |
| Identification Device | Identification |
| Forensics Examiner | ForensicsExaminer |
| Imaging | Imaging |
| Integrity | Integrity |
| Hash Function | Hashing |
| Acquired Data | AcquiredData |
| Forensic Tool | ForensicTool |
| Storage Media | ExternalStorage |
| Crime | Crime |
| Presentation | Presentation |
| Result | Result |
| Audience | Audience |
| Jury | Jury |
| Court | CourtOfLaw |
| Examination | ExaminationData |
| Analysis | AnalysisData |
| ***Model 6*** |  |
| ForensicExaminer | ForensicExaminer |
| MobileDevice | MobileDevice |
| Identification | Identification |
| Search Warrant | SearchWarrant |
| Securing Scene | SecuringScene |
| Acquisition Method | AcquisitionMethod |
| Forensic Specialist | ForensicSpecialist |
| Forensic Laboratory | ForensicLaboratory |
| Potential Evidence | PotentialEvidence |
| Procedure | Procedure |
| Imaging | Imaging |
| Recovering | RecoveringData |
| Forensic Tool | ForensicTool |
| Manual Extraction | ManualAcquisition |
| Equipment | Equipment |
| Extraction | Extraction |
| KeywordSearch | KeywordSearch |
| Extraction | ExtractionData |
| Interpretation | Interpretation |
| Copy of Evidence | Backup |
| Validation | Validation |
| Suspect | Suspect |
| Analysis | AnalysisData |
| Verification | Verification |
| Internal Memory | InternalMemory |
| Logical Acquisition | LogicalAcquisition |
| Physical Acquisition | PhysicalAcquisition |
| Hash Value | Hashing |
| Application and File Analysis | ApplicationAndFileAnalysis |
| Timeframe Analysis | TimeframeAnalysis |
| Data Hiding Analysis | DataHidingAnalysis |
| First Responder | FirstResponder |
| Environment | EnvironmentalEffect |
| Integrity | Integrity |
| Collection | ***Not supported (****add to class* ***Preservation)*** |
| Witness | Witness |
| Temperature | Temperature |
| Humidity | Humidity |
| Physical shock | Shock |
| Search Plan | Planning |
| Authorization | Authorization |
| Investigator | Investigator |
| Packaging | Packaging |
| Transporting | Transporting |
| Documentation | Documentation |
| Chain of Custody | ChainOfCustody |
| Recording | Recording |
| Court of Law | CourtOfLaw |
| Photographing | Photographing |
| Isolation | Isolation |
| Scene | Crime Scene |
| Faraday Cage | Faraday Bag |
| Evidence | Evidence |
| Law Enforcement | LawEnforcement |
| Acquired Data | AcquiredData |
| Filtering | DataFiltering |
| Removable Media | ExternalStorage |
| Incident | Crime |
| Authority | LegalAuthority |
| ***Model 7*** |  |
| Mobile Device | MobileDevice |
| Victim | Victim |
| Forensic Laboratory | ForensicLab |
| Collection | ***Not supported (****add to class* ***Preservation)*** |
| People | People |
| Crime Scene | CrimeScene |
| Procedure | Procedure |
| Legislation Authority | LegalAuthority |
| Search Warrant | SearchWarrant |
| Crime | Crime |
| Evidence | Evidence |
| Court | CourtOfLaw |
| Forensic Investigator | Investigator |
| Preparation | Preparation |
| Evidence Extraction | Extraction |
| Recording | Recording |
| Result | Result |
| Temperature | Temperature |
| Storing Evidence | TransportingAndStorage |
| Copying | Backup |
| Suspect | Suspect |
| Forensic Strategy | InvestigationStrategy |
| Planning | Planning |
| Forensic Tool | ForensicTool |
| Analysis | AnalysisData |
| Identification | Identification |
| Reconstruction | ReconstructionEvent |
| Examination | ExaminationData |
| ***Model 8*** |  |
| Preparation | Preparation |
| Crime | Crime |
| SearchWarrant | SearchWarrant |
| Forensic Tool | ForensicTool |
| Authorization | Authorization |
| Crime Scene | CrimeScene |
| Investigator | Investigator |
| Strategy | Investigation Strategy |
| Potential Evidence | PotentialEvidence |
| Securing the Scene | SecuringScene |
| Chain of Custody | ChainOfCustody |
| People | People |
| Integrity | Integrity |
| Recognition | Identification |
| External Storage Media | ExternalStorage |
| Documenting the Scene | Documentation |
| Photographing | Photographing |
| Sketching | Sketching |
| Recording | Recording |
| KeywordSearch | KeywordSearch |
| Victim | Victim |
| Suspect | Suspect |
| Witness | Witness |
| Communication Shielding | Isolation |
| Collection | ***Not supported (****add to class* ***Preservation)*** |
| Search Plan | Planning |
| Volatile Evidence | VolatileEvidence |
| Non-volatile Evidence | NonvolatileEvidence |
| Procedure | Procedure |
| Evidence | Evidence |
| Packaging | Packaging |
| Transportation | TransportationAndStorage |
| Storage | TransportationAndStorage |
| Shock | Shock |
| Temperature | Temperature |
| Examination | ExaminationData |
| Forensic Specialist | ForensicSpecialist |
| Data Filtering | DataFiltering |
| Validation | Validation |
| Humidity | Humidity |
| Evidence Bag | FaradayBag |
| Pattern Matching | PatternMatching |
| Analyzing Hidden Data | HiddenDataAnalysis |
| Reconstructing Data | ReconstructingEvent |
| Timeframe Analysis | TimeframeAnalysis |
| Application and File Analysis | ApplicationAndFileAnalysis |
| Presentation | Presentation |
| Audience | Audience |
| Law Enforcement Official | LawEnforcement |
| Technical Expert | TechnicalExpert |
| Court of Law | CourtOfLaw |
| Review | Review |
| Result | Result |
| Copy of Evidence | Backup |
| Jury | Jury |
| Legal Expert | Legal Expert |
| Analysis | AnalysisData |
| ***Model 9*** |  |
| Chain of Custody | ChainOfCustody |
| Integrity | Integrity |
| Mobile Device | MobileDevice |
| Forensic Tool | ForensicTool |
| Investigator | Investigator |
| Evidence | Evidence |
| Court | CourtOfLaw |
| Analysis | AnalysisData |
| Crime | Crime |
| Internal Memory | InternalMemory |
| External Memory | ExternalMemory |
| Conclusion | Conclusion |
| Collection | ***Not supported*** *(add to* ***Preservation*** *class)* |
| Examination | ExaminationData |
| Law Enforcement | LawEnforcement |
| Search Warrant | SearchWarrant |
| Cut Off All the Wireless Networks | Isolation |
| Imaging | Imaging |
| Hashing | Hashing |
| KeywordSearch | KeywordSearch |
| Forensic Lab | ForensicLab |
| Identification | Identification |
| Hypothesis | Hypothesis |
| Recovering Data | RecoveringData |
| Presentation | Presentation |
| Result | Result |
| Forensic Examiner | ForensicExaminer |
| Audience | Audience |
| Documentation | Documentation |
| ***Model 10*** |  |
| Presentation | Presentation |
| Review | Review |
| Acquisition Method | AcquisitionMethod |
| Smartphone | Mobile Device |
| Integrity | Integrity |
| Internal Memory | InternalMemory |
| Forensic Investigator | Investigator |
| Examined Data | ExaminedData |
| Collection | ***Not supported (****add to class* ***Preservation)*** |
| Forensic Tool | ForensicTool |
| Examining Data | ExaminingData |
| Authentication | Authentication |
| Court | CourtOfLaw |
| Preparation | Preparation |
| Securing the Scene | Securing Scene |
| Chain of Custody | ChainOfCustody |
| Recognition | Identification |
| Documenting the Scene | Documentation |
| Communication Shielding | Isolation |
| Volatile Evidence | VolatileEvidence |
| Non-Volatile Evidence | NonVolatileEvidence |
| Evidence | Evidence |
| Recovering Data | RecoveringData |
| Crime Scene | CrimeScene |
| Examination Data | ExaminationData |
| Analysis | AnalysisData |
